# Cadmium removal and indole acetic acid production by ureolytic bacteria isolated from rhizosphere soils

**DOI:** 10.1007/s11274-025-04482-9

**Published:** 2025-08-08

**Authors:** Carlos A. Adarme-Duran, Elianna Castillo, Pedro F. B. Brandão

**Affiliations:** 1 Facultad de Ciencias, Instituto de Biotecnología - IBUN, Universidad Nacional de Colombia - sede Bogotá, Carrera 30 # 45-03, Bogotá, Colombia; 2 Facultad de Ciencias, Departamento de Química, Grupo de Estudios para la Remediación y Mitigación de Impactos Negativos al Ambiente (GERMINA), Universidad Nacional de Colombia - sede Bogotá, Carrera 30 # 45- 03, Bogotá, Colombia

**Keywords:** Bioremediation, Cd-tolerant bacteria, Cocoa, Heavy metal, IAA production, MICP.

## Abstract

This work reports the isolation of 54 ureolytic bacteria with microbiologically induced carbonate precipitation (MICP) activity from *Theobroma cacao* L. rhizosphere soils with Cd concentration ranging from 0.49 to 21.29 mg kg^− 1^. Analysis of the 16 S rRNA gene showed the isolates belong to the genera *Serratia*, *Pseudomonas*, *Comamonas*, *Klebsiella*, *Bacillus*, *Citrobacter*, *Flavobacterium*, *Delftia*, and *Stenotrophomonas*. They showed ureolytic activity between 0.31 and 1.01 µmol NH_4_^+^ mL^− 1^ h^− 1^ and produced the phytohormone indole acetic acid (IAA). Interestingly, Cd was observed to increase ureolytic activity and did not affect the IAA production of various isolates. Cadmium removal in solution ranged from 4.4 to 87.0% after 48 h. Strains *Serratia* sp. 89a, *Klebsiella* sp. 76 h, and *Stenotrophomonas* sp. 67w exhibited the best performance (> 80%). Cadmium removal through MICP by the genera *Delftia*, *Flavobacterium*, and *Klebsiella* is reported for the first time. Results suggest the isolated bacteria have the potential for Cd bioremediation and could be used to alleviate metal-induced stress through IAA production.

**Supplementary Information:**

The online version contains supplementary material available at 10.1007/s11274-025-04482-9.

## Introduction

Cadmium is a trace element naturally present in soils with an estimated global average of 0.36 mg kg^− 1^ (Kubier et al. 2019). Its concentration can increase due to geogenic sources, such as the weathering of parent material (e.g., black shales), and anthropogenic activities, such as the use of wastewater or fertilizers (e.g., phosphate rock), in the case of agricultural soils (Kubier et al. 2019). Particularly, in cacao-growing soils it has been pointed out that Cd arises mainly from natural processes (Vanderschueren et al. 2021). An important aspect of Cd in soils is its mobility. Dissolved cadmium generally occurs as the divalent cation Cd^2+^, and forms water-soluble complexes with anions (e.g., Cl^−^) and dissolved organic matter, thus remaining in solution (Kubier et al. 2019). Therefore, Cd can be absorbed by plants and reach humans through the food chain. Exposure to Cd is of concern because it can cause health problems such as different types of cancer, osteoporosis, and oxidative stress, responsible for liver and kidney diseases (Genchi et al. 2020). Hence, it is crucial to develop strategies to remediate Cd in agricultural soils.

Remediation of metal-contaminated soils by bioprecipitation reduces the mobility of toxic elements and is stable under different environmental conditions (Liu et al. 2021). Microbiologically induced carbonate precipitation (MICP) is an emerging and efficient strategy to reduce the availability of contaminating metals (Zheng et al. 2021; Tamayo-Figueroa et al. 2019). MICP through the ureolytic pathway is the most studied, standing out for its high efficiency (Zhang et al. 2023). In this mechanism, urea is hydrolyzed generating carbonate and ammonium ions, and promoting the alkalinization of the medium, which ultimately induces the precipitation or co-precipitation of divalent metals (e.g., Cd^2+^) (Torres-Aravena et al. 2018). By applying bacteria with MICP activity, a reduction in Cd mobility has been demonstrated. Studies in rice cultivation have reported a decrease in the exchangeable fraction of Cd and an increase in the more stable carbonated fraction (Yang et al. 2024; Cai et al. 2023). Thus, metal absorption in rice and Asian cabbage has been reduced (Yan et al. 2024; Zhou et al. 2021).

The use of an appropriate bacterial strain with a suitable methodology could not only contribute to the immobilization of toxic elements through the MICP process but could also mitigate the adverse effects of these elements on plant growth. It has been found that bacteria with MICP activity isolated from the rhizosphere of plants, can produce the phytohormone indole acetic acid (IAA) (Jalilvand et al. 2019), although this has been little explored. *Bacillus thuringiensis* X30, a IAA-synthesizing, urease-producing bacterium isolated from the rhizosphere soil of *Amaranthus tricolor*, demonstrated dual functionality in Cd remediation: reduced Cd availability and its uptake by Potato tubers (*Solanum tuberosum* L. Zhongshu-3) and concurrently increase plant biomass (Cheng et al. 2020; Han et al. 2018). Evidencing plant-growth promoting traits (e.g., IAA production) and Cd immobilization (e.g., via MICP activity) in rhizobacteria is critical to develop comprehensive bioremediation strategies, thereby leveraging bacterial multifunctionality to address both soil and plant components of contamination (Halim et al. 2020).

The presence of Cd in cacao-growing soils in Colombia has been reported in different regions, particularly affecting Santander, the largest producer nationwide, due to the presence of hotspots (Bravo et al. 2021). Bacteria with MICP activity with potential for Cd bioremediation have been isolated from cacao-growing soils in this region (Diez-Marulanda and Brandão 2023). However, to the best of our knowledge, the isolation of bacteria from *Theobroma cacao* L. rhizosphere soils, which may have better MICP performance than those isolated from non-rhizosphere soils (Diez-Marulanda and Brandão 2024), due to higher Cd levels and ureolytic activity when comparing both types of soils (Adarme-Duran et al. 2024), has not been explored. In this work, bacteria were isolated from the rhizosphere of cacao plants, with the aim of finding bacteria with MICP activity and IAA production. Ureolytic bacteria were isolated and their MICP activity was verified on solid medium. The ability of bacteria with MICP activity to produce the phytohormone IAA was evaluated. Isolates were identified by 16 S rRNA gene analysis, and the effect of Cd on ureolytic activity and IAA production was investigated. Finally, the ability of the bacterial isolates to remove Cd in solution was determined. At least 17 bacteria with potential for application in cadmium bioremediation in cacao-growing soils are reported.

## Materials and methods

### Chemical reagents

All reagents used were of analytical grade. Stock solutions of Cd(II) (10 g L^− 1^), Ca(II) (27 g L^− 1^) and urea (500 g L^− 1^) were prepared from CdCl_2_.H_2_O (Merck, Germany), CaCl_2_.2H_2_O (PanReac AppliChem, Germany) and urea (ChemCruz, USA), respectively. These solutions were sterilized by filtration (0.22 μm, Sartorius, Germany) before being added, under aseptic conditions, to the sterile culture media. The culture media was sterilized by autoclave (121 °C, 15 psi, 30 min).

### Sampling of Cacao rhizosphere soils

Cacao rhizosphere soil samples were collected from two farms (*La Perla* and *Los Cedros*) located at El Carmen de Chucurí - Santander – Colombia (Adarme-Duran et al. 2024). The study area was previously evaluated, and it was determined that the rhizosphere soils have Cd concentrations between 0.27 and 21.29 mg kg^− 1^ (Adarme-Duran et al. 2024).

To collect the soil samples, the cacao plant roots were carefully taken and manually shaken to remove the non-rhizosphere soil. Root-adhering soil, up to a distance of 10 mm (rhizosphere soil; Kuzyakov and Razavi 2019) was collected and stored in sterile conical tubes (15 mL) under refrigerated conditions (4 °C) in portable refrigerators.

### Isolation of Cd-tolerant ureolytic bacteria

To isolate Cd-tolerant bacteria, 16 soils from both farms were selected, using Cd concentration and geographical location within each farm as criteria. Following this strategy, 12 soils from *La Perla* farm and 4 soils from *Los Cedros* farm were used. Based on cadmium concentrations, the soils were categorized as follows: three soils contained Cd levels greater than 7 mg kg⁻¹, four soils had Cd concentrations between 2 and 7 mg kg⁻¹, and nine soils exhibited Cd levels below 2 mg kg⁻¹. Metal concentrations were determined previously (Adarme-Duran et al. 2024) using 8 mL of *aqua regia* (HCl: HNO_3_ 3:1) heated in a graphite block digestion system (DigiPREP, SCP SCIENCE, Canada) with the following temperature program: room temperature increase to 120 °C with a temperature holding time of 120 min at 120 °C, and Cd in solutions were analyzed by flame atomic absorption spectrophotometry. To isolate the bacteria, 1 g of soil was suspended in 9 mL of NaCl (0.85% m/v) and stirred for 6 h on a rotary shaker at room temperature. Serial dilutions of the supernatant were made up to 10^− 7^ with NaCl (0.85% m/v) and spread by extension on Petri dishes with a modified Christensen selection medium (Qiao et al. 2021; Christensen 1946), composed of tryptose (5 g L^− 1^), meat peptone (10 g L^− 1^), urea (20 g L^− 1^), NaCl (5 g L^− 1^), phenol red (0.012 g L^− 1^), Cd(II) (5 mg L^− 1^) and agar (15 g L^− 1^) (Medium A). The cultures were incubated at 30 °C for 1 to 2 days until colonies were observed. Uninoculated medium was used as negative control during bacterial isolation to ensure the absence of contamination. The colonies were selected based on the color change of the medium from yellow to pink (this color change is associated with alkalinization of the medium due to urea degradation; Achal et al. 2011) and pure cultures were obtained by repetitive sowing on the same medium. This procedure allows the selection of ureolytic bacteria; however, it does not indicate the MICP activity of the isolates.

### Screening of MICP activity of the ureolytic bacteria

The MICP activity of the selected ureolytic bacteria was qualitatively examined by spreading pure cultures on Petri dishes with a culture medium composed of tryptose (5 g L^− 1^), meat peptone (10 g L^− 1^), urea (20 g L^− 1^), NaCl (5 g L^− 1^), Ca(II) (1.2 g L^− 1^), Cd(II) (5 mg L^− 1^) and agar (15 g L^− 1^) (Medium B). The cultures were incubated at 30 °C for 1 to 6 days to allow the precipitate formation and growth. To verify the presence of precipitates, the cultures were observed with the naked eye and using an optical microscope (CX31 Upright Microscope, Olympus) coupled to a digital camera (CMOS Sensor, Aptina). Considering that precipitates feel hard to the touch, a test was performed with a metal inoculating loop. Additionally, the presence of carbonates was determined using HCl (1.2 M), since CO_2_ is generated and appears as effervescence (Montoya et al. 2005). As a negative control for precipitate formation, the isolates were spread on Medium B without urea and calcium, which does not promote carbonate precipitation. Uninoculated medium was used as negative control to rule out abiotic precipitation and contamination. Pure cultures were cryopreserved in liquid medium (Tryptose (5 g L^− 1^), meat peptone (10 g L^− 1^), NaCl (5 g L^− 1^), supplemented with urea (20 g L^− 1^) and glycerol (20% v/v) at -80 °C.

### Phenotypic characterization of ureolytic bacteria

To characterize the isolates, Gram staining was performed (Sanders and Miller 2010) and the morphology was observed under an optical microscope (CX31 Upright Microscope, Olympus) coupled to a digital camera (CMOS Sensor, Aptina). The colony morphology was determined (Breakwell et al. 2007) on LB-agar medium (Tryptose 10 g L^− 1^, yeast extract 5 g L^− 1^, NaCl 10 g L^− 1^, agar 15 g L^− 1^) supplemented with Cd(II) (5 mg L^− 1^) and incubated at 30 °C, for 24–48 h.

### Molecular identification of ureolytic isolates

To carry out the molecular identification of the isolates, cultures incubated at 30 °C for 24–48 h were used. To obtain genomic DNA, the Wizard Genomic DNA Purification kit (Promega Co., USA) was used following the supplier’s recommendations. In some cases, cell lysis was assisted by bead-beating (Mini-Beadbeater-16, BioSpec Products, USA) using glass disruptor beads (0.5 mm, USA Scientific) with two 30-second beating cycles and 1-min rest on ice between cycles. Once the genomic DNA of the isolates was obtained, a single-stranded conformation polymorphism (SSCP) analysis of the V4-V5 regions of the 16 S rRNA gene was performed to dereplicate the isolates (Brandão et al. 2002). Polymerase chain reaction (PCR) was performed using primers 519 F and 909R **(**Supplementary Table S1) to amplify the V4-V5 regions (Schwieger and Tebbe 1998; Kato et al. 1997). Molecular identification was performed by amplification of the 16 S rRNA gene using primers 27 F and 1492R (Supplementary Table S1).

Each PCR was performed in a total volume of 20 µL using 1 µL of template DNA in a Master Mix as described in Supplementary Table S2. The temperature program used for amplification was as follows: initial cycle at 94 °C for 5 min; 30 amplification cycles (denaturation at 94 °C for 20 s, annealing at 55 °C for 20 s, and elongation at 72 °C for 40 s); and finally, a cycle at 72 °C for 5 min (C1000 Thermal Cycler, Bio-Rad). Amplicons size was determined by 1.2% (m/v) agarose gel electrophoresis (HyAgaroseTM, HydraGene, China), stained with SYBR Safe (Invitrogen, USA), and using a 1 kb ExcelBand 1 marker (SMOBIO) or 100 bp plus DNA Ladder (BIORON). Finally, the gel was photodocumented (Safe Imager™ 2.0 Blue-Light Transilluminator, Invitrogen). Ammonium acetate (5 M) and absolute ethanol were used for amplicon purification. Briefly, 20 µL of the PCR products were mixed with 2 µL of the ammonium acetate solution and 40 µL of cold absolute ethanol. Subsequently, the mixture was shaken and centrifuged at 15,000 rpm at 4 °C for 20 min. After successive washes with ethanol, the pellet was dried and rehydrated in Milli-Q water overnight and stored at − 20 °C. The concentration and quality of the amplicons was determined by spectrophotometry (NanoDrop 2000c, Thermo Fisher Scientific).

The SSCP analysis was performed as previously reported (Montaño-Salazar et al. 2018; Brandão et al. 2002) in 0.7X MDE at 5 W of constant power for 16 h 30 min, and the gel was stained with silver nitrate (Bassam et al. 1991). Based on phenotypic characterization, ureolytic activity, IAA production, and differentiated SSCP profiles, 40 isolates were selected for molecular identification. The purified PCR products were commercially sequenced using the Sanger method (SSiGMol – Instituto de Genética – Universidad Nacional de Colombia). The sequences were edited with BioEdit software (Hall 1999) version 7.2.5, deposited in GenBank (accession numbers PQ497356 to PQ497395), and analyzed by BLASTn (https://blast.ncbi.nlm.nih.gov/Blast.cgi). Sequence alignments were performed using Muscle (Edgar 2004) in MEGAX software (Kumar et al. 2018) with default parameters for phylogenetic analysis by bayesian inference using MrBayes v 3.2.7. (Ronquist et al. 2012). The nucleotide substitution model GTR + G + I (G: 5 categories) was previously selected with JmodelTest v 2.1.10 (Darriba et al. 2012; Guindon et al., 2003). A total of 1,500,000 generations were produced, and the tree was selected by majority consensus. The results obtained were evaluated with Tracer v 1.7.2. The selected tree was visualized and edited with the FigTree v1.4.4 program (http://tree.bio.ed.ac.uk/software/figtree/). The 16 S rRNA gene from bacterium *Thermus aquaticus* Y51MC23 (GenBank accession number: CP010822.1) was used as an outgroup.

### Determination of ureolytic activity and IAA production by Cd-tolerant bacteria

To determine ureolytic activity and IAA production, a 24–48 h liquid medium culture in LB was used, and the biomass was adjusted according to the optical density at 600 nm (OD_600_) to approximately 0.3 with NaCl (0.85% m/v) prior to experiments. Then, 0.1 mL of the bacterial suspension was inoculated into 10 mL (1% v/v) of culture medium in an amber bottle. Each assay was performed in triplicate.

For ureolytic activity, the medium used was LB (tryptose (10 g L^− 1^), yeast extract (5 g L^− 1^), NaCl (10 g L^− 1^) supplemented with urea (20 g L^− 1^), with an incubation period of 48 h at 30 °C and 120 rpm. An aliquot was then taken and centrifuged at 15,000 rpm for 10 min. Urease activity was quantified according to ammonium production using the indophenol blue method based on a modified Berthelot reaction (Verdouw et al. 1978). Briefly, an aliquot of the supernatant (diluted with deionized water) was mixed with 160 µL of colorimetric agent (sodium salicylate (0.50 M) and sodium nitroprusside (0.02 M), 80 µL of buffer solution (sodium hydroxide (0.74 M), sodium monohydrogen phosphate Na₂HPO₄0.7 H₂O (0.37 M) and sodium hypochlorite (10% v/v), pH 13), and 40 µL of chelating agent (Na_2_EDTA solution (0.15 M) to prevent cation precipitation. Color development was carried out for 30 min at 37 °C. Quantification was performed using an external calibration curve (0.17–2.08 mg L^− 1^) prepared with an ammonium standard (Merck, 1000 mg L^− 1^) and the absorbance was measured at 680 nm on a NanoDrop 2000c (Thermo Scientific). *Bacillus thuringiensis* was used as a positive control and the culture medium without bacteria was used as a negative control.

To determine IAA production, LB medium supplemented with L-tryptophan (1 g L^− 1^) was used. Incubation was carried out for 72 h at 30 °C and 120 rpm. The quantification of the IAA produced was carried out colorimetrically using Salkowsky’s reagent (Gang et al. 2019). After the incubation period, an aliquot was taken and centrifuged at 15,000 rpm for 10 min. Then, 1 mL of the supernatant (diluted in deionized water) was taken, mixed with 1 mL of Salkowsky’s reagent (1 mL FeCl_3_ (0.5 M) + 49 mL HClO_4_ (35% v/v) and incubated at 30 °C for 30 min in the dark. Finally, IAA was quantified at 530 nm on a NanoDrop 2000c, using an IAA (Alfa Aesar) calibration curve (2–40 mg L^− 1^). *Azospirillum brasiliense* was used as a positive control and the culture medium without bacteria was used as a negative control.

### Effect of Cd on ureolytic activity and IAA production

The effect of Cd on ureolytic activity and IAA production was carried out in only 6 bacterial isolates selected according to the range of values previously found for enzymatic activities: 2 bacteria with high values, 2 bacteria with low values, and 2 bacteria with intermediate values. Additionally, the bacteria genus was considered as a selection criterion since the impact of Cd on enzymatic activities may change for different genera (Diez-Marulanda and Brandão 2024; Peng et al. 2020; Carlos et al. 2016; Dell’Amico et al. 2008).

To determine the effect of Cd on ureolytic activity and IAA production, 24-h-old cultures (OD_600_ ≈ 0.3) were inoculated at 1% (v/v) in 10 mL of the corresponding culture medium in the absence and presence of Cd. For ureolytic activity, LB medium in the presence and absence of Cd were used: (1) LB supplemented with urea (20 g L^− 1^) and (2) LB + urea (20 g L^− 1^) + Cd(II) (60 mg L^− 1^). For IAA production, LB culture medium (1) supplemented with L-tryptophan (1 g L^− 1^) and (2) LB + L-tryptophan (1 g L^− 1^) + Cd (60 mg L^− 1^) were used. Incubation conditions and quantification for both enzymatic activities were described previously (ureolytic activity and IAA production assays). The tests were carried out in triplicate. In this case, the enzymatic activity values were divided by the colony forming units (CFU) determined at the end of the assay, because of Cd effect on bacterial growth. The Cd concentration used in the experiments was selected so that it would not be a limiting factor in future soil applications and considering a possible reduction in MICP performance of the isolates when applied to soils compared to solution tests (Peng et al. 2020). Thus, the Cd concentration was chosen to correspond to twice the maximum Cd concentration found in cacao soils (27 mg kg^− 1^) reported for the studied area (Bravo et al. 2021).

### Cd removal in solution

To determine the bacteria’s Cd removal, 10 mL of LB medium supplemented with urea (20 g L^− 1^), Ca(II) (1.2 g L^− 1^) and Cd(II) (60 mg L^− 1^) were inoculated with 0.1 mL of bacterial suspension (OD_600_ ≈ 0.3, 24-h-old culture) and incubated for 48 h at 30 °C and 120 rpm. An experimental negative control (medium without bacteria) of Cd removal was performed. The culture was then vortexed and an aliquot was taken to determine the OD_600_. The remaining culture medium was centrifuged at 15,000 rpm for 10 min. Three aliquots of the supernatant were taken to measure pH (Oakton pH-meter), quantify the ammonium produced (described previously) and quantify Cd. To determine the latter, aliquots were treated with 5 mL of *aqua regia* (3 HCl: 1 HNO_3_) and brought to final volume with HNO_3_ (0.5% v/v). The samples were analyzed by flame atomic absorption spectrophotometry (ContrAA 700 - High-Resolution Continuum Source, Analytik Jena), and quantified by external calibration on a curve prepared with a Cd standard solution (Panreac, 1000 mg L^− 1^). All assays were performed in triplicate. The concentration of Cd in the control (Cd_control_) and in the medium inoculated with each bacterium (Cd_bacteria_) were used to calculate, according to Eq. 1, the percentage of removed Cd (%R Cd) at the end of each assay.

1 $$\:\%R\:Cd=\:\frac{{Cd}_{control}-\:{Cd}_{bacteria}}{{Cd}_{control}}\:x\:100\%\:$$

### Statistical analysis

Data analysis was performed using R software (version 4.2.2, R Core Team 2022), RStudio (version 2022.12.0 + 353; RStudio Team 2022). The experiments were performed in triplicate and the results are presented as the average. Standard error (Cumming et al. 2007) and t-test were used to determine significant differences between ureolytic activity and IAA production of the selected bacteria in culture media with and without Cd.

## Results

### Isolation and identification of Cd-tolerant ureolytic bacteria

**Fig. 1:**
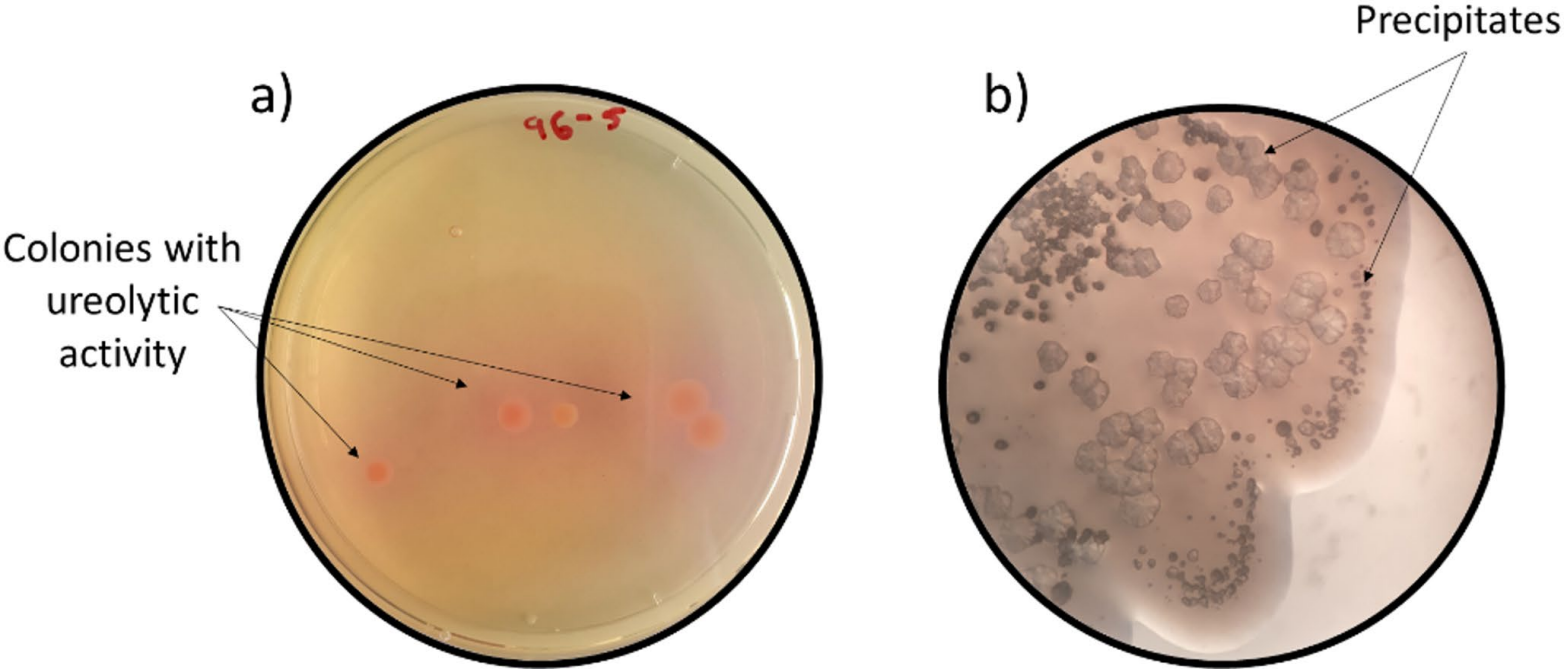
Bacteria isolated from cacao rhizosphere soils. Qualitative screening of **(a)** Colonies with ureolytic activity showing a pink color on urea-agar supplemented with Cd(II) (5 mg L^− 1^) and **(b)** Precipitates formed by MICP activity on urea-agar supplemented with Cd(II) (5 mg L^− 1^) and Ca(II) (1.2 g L^− 1^), observed under an optical microscope (CX31 Upright Microscope, Olympus)

Through a two-step selection process, 54 isolates with ureolytic and MICP activities were obtained. In the first isolation step, colonies with the ability to hydrolyze urea (ureolytic activity) were identified according to the pink color observed in Medium A (Fig. 1a). Subsequently, these isolates were spread in Medium B that induces the formation of carbonate precipitates to qualitatively verify the MICP activity (Fig. 1b). Both steps permitted to qualitatively observe differences in the ureolytic activity (indicated by color intensity) and the MICP activity (through precipitate sizes and shapes) of the isolates (Supplementary Fig. S1). The selected colonies showed a great variety in shape, size, texture, margin and color (Supplementary Table S3); 38 out of the 54 isolates were gram-negative bacteria, and the microscopic morphology was primarily rod-shaped bacteria (Supplementary Table S4).

Based on SSCP analysis of the V4-V5 regions of the 16 S rRNA gene, where fragments with different sequences adopt spatial conformations that result in differentiated electrophoretic profiles (Schwieger and Tebbe 1998), dereplication of isolates was carried out, finding 38 different profiles (Supplementary Fig. S2). Considering this analysis and other characteristics (morphology, ureolytic activity and IAA production), 16 S rRNA gene sequencing was performed for 40 isolates. In some cases, the 16 S rRNA gene was sequenced for isolates that presented the same SSCP profile, due to relevant differences in the other characteristics studied. According to BLAST analysis, 9 genera were identified with the number of isolates indicated in parentheses: *Serratia* (2), *Pseudomonas* (14), *Comamonas* (1), *Klebsiella* (2), *Bacillus* (13), *Citrobacter* (4), *Flavobacterium* (1), *Delftia* (1) and *Stenotrophomonas* (2) (Supplementary Table S5). Phylogenetic analysis by bayesian inference showed concordance with the results obtained by BLAST. The strain genera previously identified by BLAST were grouped into the corresponding clades with posterior probability > 0.97 (Supplementary Fig. S3). For some bacteria, the genus was assigned according to SSCP profile (Supplementary Table S6), for example, the genus *Klebsiella* was assigned to isolate 76 h since it has the same SSCP profile as isolates 76b and 76c2 (Supplementary Fig. S2), both identified as *Klebsiella* according to 16 S rRNA gene analysis (Supplementary Table S5).

### Quantitative ureolytic activity and production of IAA

**Fig. 2:**
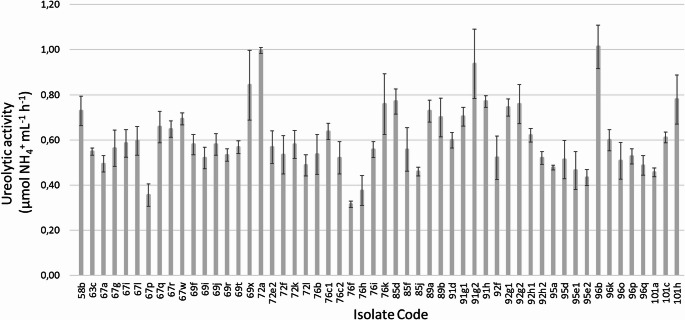
Ureolytic activity of isolated bacteria. Evaluation by ammonium (NH_4_^+^) production in 48 h at 30 °C and 120 rpm using LB medium supplemented with urea (20 g L^− 1^). Error bars indicate 2*SE (*n* = 3)

The bacteria showed ureolytic activity ranging from 0.31 to 1.01 µmol NH_4_^+^ mL^− 1^ h^− 1^ (Fig. 2), where the highest values correspond to 3 isolates of the *Bacillus* genus (strains 96b, 72a and 91g2). In contrast, *Comamonas* sp. 76f had the lowest enzymatic activity. It is worth noting that for some genus, we found a relevant variation of ureolytic activity. For instance, the isolates belonging to *Bacillus* presented values between 0.52 and 1.01 µmol NH_4_^+^ mL^− 1^ h^− 1^ (% coefficient of variation = 17.9). *Pseudomonas* strains showed a lower, although important, variation from 0.46 to 0.65 µmol NH_4_^+^ mL^− 1^ h^− 1^ (% coefficient of variation = 8.3). *Klebsiella* isolates has ureolytic activities between 0.38 and 0.51 µmol NH_4_^+^ mL^− 1^ h^− 1^. For *Citrobacter*, *Serratia* and *Stenotrophomonas* isolates, we found similar ureolytic activities between the same genera.

**Fig. 3:**
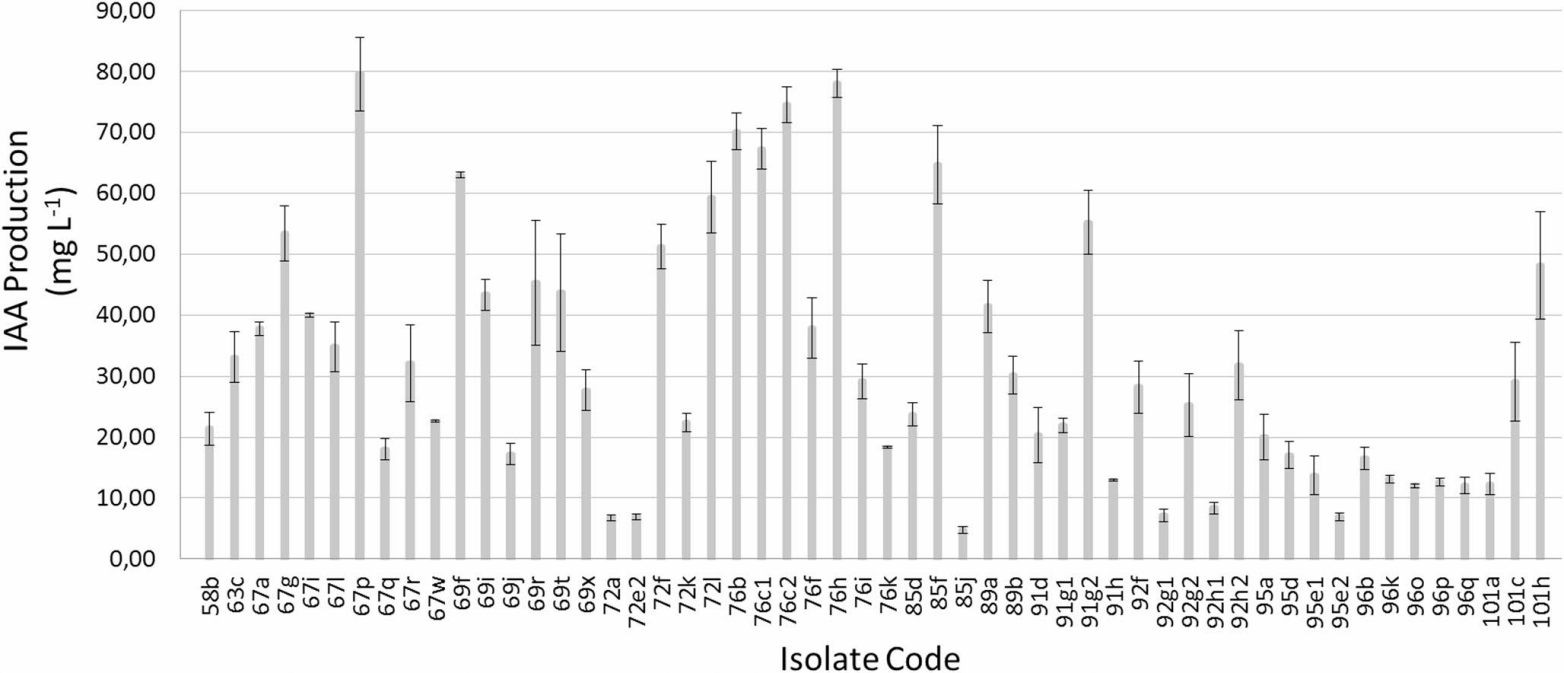
IAA production by isolated bacteria after 72 h at 30 °C and 120 rpm in LB medium supplemented with L-tryptophan (1 g L^− 1^). Error bars indicate 2*SE (*n* = 3)

In this study, IAA production was observed in all isolated bacteria, with mean values between 4.8 and 79.5 mg L^− 1^ (Fig. 3). Isolates *Klebsiella* sp. 76 h, *Delftia* sp. 67p and *Klebsiella* sp. 76c2, showed the highest IAA production, while *Pseudomonas* sp. 85j had the lowest. IAA concentrations varied widely among *Bacillus* and *Pseudomonas*, ranging from 6.7 to 67.3 mg L^− 1^ and from 4.8 to 64.6 mg L^− 1^, respectively. Although a similar range was found for these genera, *Bacillus* showed a higher variation (% coefficient of variation = 69.9) than *Pseudomonas* (% coefficient of variation = 50.1). On the other hand, *Citrobacter* showed consistently lower IAA production than *Klebsiella*, *Serratia* and *Stenotrophomonas*.

### Effect of Cd on ureolytic activity and IAA production

**Fig. 4:**
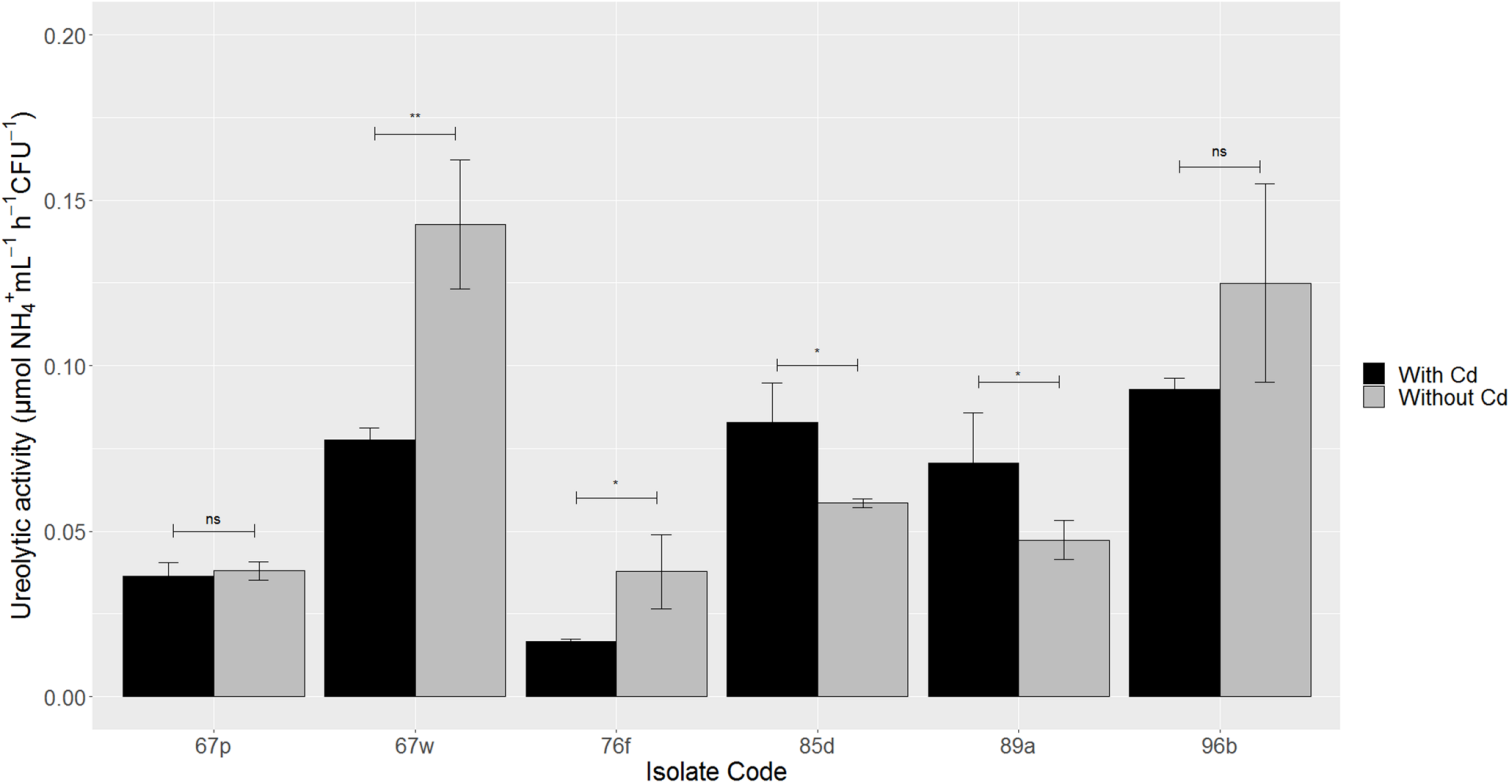
Effect of Cd(II) (60 mg L^− 1^) on ureolytic activity of selected isolates. Error bars indicate 2*SE (*n* = 3). ns: non-significant difference, * p-value < 0.05, ** p-value < 0.01

The effect of Cd on ureolytic activity varied for the bacteria studied (Fig. 4). For isolates *Comamonas* sp. 76f and *Stenotrophonomas* sp. 67w, a reduction in ureolytic activity of 55.9% and 45.7%, respectively, was observed. For isolates *Bacillus* sp. 85d and *Serratia* sp. 89a, the presence of Cd increased ureolytic activity by 41.9% and 49.3%, respectively. However, no significant differences were observed for isolates *Delftia* sp. 67p and *Bacillus* sp. 96b. It was observed a different effect of Cd on the ureolytic activity of the two *Bacillus* strains evaluated. The presence of Cd (60 mg L^− 1^) reduced *Bacillus* sp. 96b enzymatic activity by 26%, although this was statistically insignificant. In contrast, the metal increased the ureolytic activity of *Bacillus* sp. 85d by 42%.

**Fig. 5:**
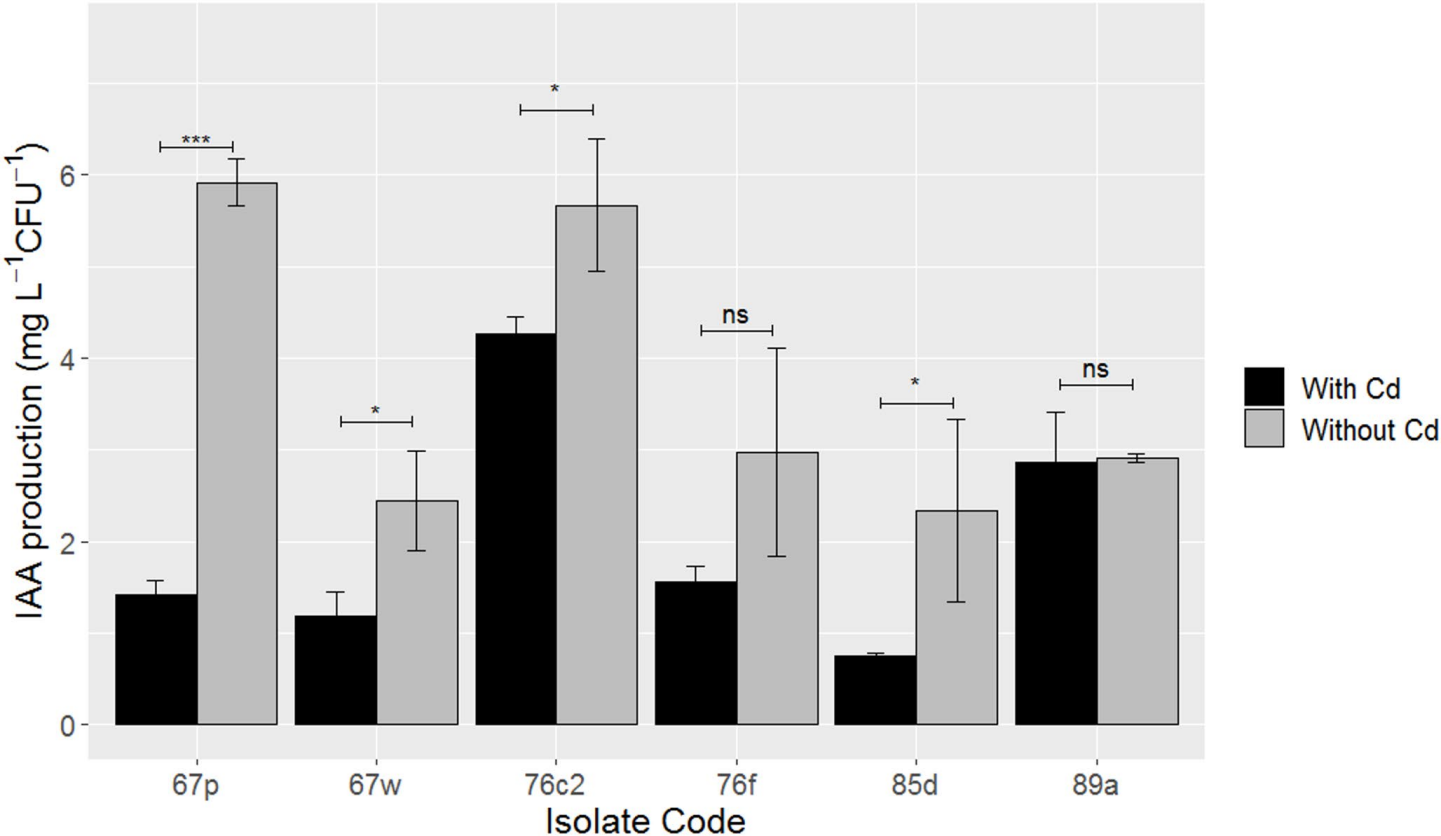
Effect of Cd(II) (60 mg L^− 1^) on IAA production for selected isolates. Error bars indicate 2*SE (*n* = 3). ns: non-significant difference, * p-value < 0.05, *** p-value < 0.001

The presence of Cd caused a negative effect on the IAA production of some bacteria (Fig. 5). For isolates *Delftia* sp. 67p, *Stenotrophomonas* sp. 67w, *Klebsiella* sp. 76c2 and *Bacillus* sp. 85d, the presence of Cd decreased IAA production by 76,0%, 51,8%, 24,7% and 67,6%, respectively. Although all four strains exhibited significant reductions, the effect of Cd was notably stronger in *Delftia* sp. 67p, *Stenotrophomonas* sp. 67w and, *Bacillus* sp. 85d compared to *Klebsiella* sp. 76c2. In contrast, *Serratia* sp. 89a and *Comamonas* sp. 76f showed no significant changes in IAA production in the presence of Cd.

### Cd removal in solution

The Cd removal percentages varied between 4.4 and 87.0% (Supplementary Table S7). The pH, ureolytic activity and OD_600_ ranged between 7.45 and 9.05 pH units, 0.11 and 1.14 µmol NH_4_^+^ mL^− 1^ h^− 1^ and 0.2–3.4, respectively. High Cd removal was observed in *Serratia* sp. 89a (87.0%), *Klebsiella* sp. 76 h (83.4%), *Stenotrophomonas* sp. 67w (82.7%), and *Serratia* sp. 58b (80.8%). Among the isolates, *Serratia* strains showed a consistently higher Cd removal. Although *Stenotrophomonas* sp. 67w had a higher Cd removal percentage, the strain *Stenotrophomonas* sp. 67q presented an intermediate value of 49.2%. A similar result was observed for *Klebsiella* strains. Cd removal varied widely among *Bacillus* and *Pseudomonas*, ranging from 9.1 to 68.8% and from 4.4 to 48.9%, respectively, where *Bacillus* tend to have a higher Cd removal compared to *Pseudomonas*.

## Discussion

The isolations were made from cacao rhizosphere soils, considering they presented greater ureolytic activity and Cd concentration compared to non-rhizosphere soils (Adarme-Duran et al. 2024). This agrees with previous reports where ureolytic bacteria with MICP activity have been isolated from rhizosphere soils (Yan et al. 2024; Jalilvand et al. 2019). Furthermore, three Cd-tolerant bacteria of the *Serratia* and *Acinetobacter* genera with MICP activity were isolated from non-rhizosphere soils in the same area, and showed Cd removal percentages in solution greater than 90% from an initial 0.05 mM Cd(II) concentration (Diez-Marulanda and Brandão 2023). In this study, a relevant amount of ureolytic bacteria with MICP activity from diverse genera were recovered from cacao rhizosphere soils. Access to bacteria belonging to different genera is of interest because significant differences in the ability to immobilize toxic elements have been reported (Kumar et al. 2023). The genus *Sporosarcina* stands out in various studies, although it has been shown that other genera can have high (> 90%) removal percentages, like *Bacillus*,* Pseudomonas* and *Serratia* (Taharia et al. 2024; Fang et al. 2021). Previous studies have reported the MICP activity of the majority of genera recovered in this study: *Serratia* (Diez-Marulanda and Brandão 2023; Bhattacharya et al. 2018), *Pseudomonas* (Diez-Marulanda and Brandão 2023; Al Disi et al. 2022), *Comamonas* (Zhou et al. 2021), *Klebsiella* (Duan et al. 2023), *Bacillus* (Li et al. 2022; Bibi et al. 2018; Zhu et al. 2016), *Citrobacter* (Wei et al. 2022b), *Flavobacterium* (Ferrer et al. 1988) and *Stenotrophomonas* (Jalilvand et al. 2019). However, no report has been found for *Delftia* isolates. This genus has been mentioned in a study in which a consortium was used to induce carbonate precipitation, where microbial composition and abundance were determined using Illumina MiSeq (Okyay et al. 2016). Nevertheless, the authors mentioned that it was not possible to determine the participation of the genus *Delftia* in the MICP process (Okyay et al. 2016). No other reports evidencing MICP activity by *Delftia* genus were found, implying that this is the first one. In general, it can be mentioned that the isolates presented low ureolytic activity compared to other reports with values between 3.05 and 23.1 µmol NH_4_++ mL-1-1 min-1-1 (Al Disi et al. 2022; Qiao et al. 2021; Bibi et al. 2018). Nonetheless, the activity was in the range of other bacteria recovered from cacao-growing soils (Diez-Marulanda and Brandão 2023). Despite the low ureolytic activity, the capacity of the bacteria to precipitate carbonates was qualitatively observed.

The IAA production detected in the isolates is attractive because it could alleviate the adverse effects caused by Cd on plant development (Etesami 2018). Previous reports have determined IAA production by ureolytic bacteria. Han et al. (2020) reported IAA production values of 321 mg L^− 1^ and 413 mg L^− 1^ for *Serratia liquefaciens* and *Priestia megaterium* (syn. *Bacillus megaterium)*, respectively, comparable to those reported by Wang et al. (2020) for *Enterobacter bugandensis* TJ6 and *Bacillus megaterium* HD8, with values of 303 mg L^− 1^ and 387 mg L^− 1^, respectively. These strains have a higher IAA production than that found in this study (from 4.8 to 79.5 mg L^− 1^). Though, like our results, Wei et al. (2022b) reported for *Bacillus* sp. UR21 an IAA production of 5.45 mg L^− 1^, while Zhou et al. (2021) reported for *Comamonas testosteroni* ZG2, an IAA production of 18 mg L^− 1^. Since IAA can have positive or negative impacts on plants depending on dosage (Bunsangiam et al. 2021), the isolation of bacteria with different levels of IAA production is of relevance for field applications depending on the required amount.

After an adequate selection, the inoculation of ureolytic bacteria capable of producing IAA allowed an increase in the biomass of different plants along with a reduction of Cd absorption (Wei et al. 2022a; Han et al. 2020; Wang et al. 2020). The application of *Comamonas testosteroni* ZG2 (with MICP activity and IAA production) mitigated the presence of Cd and increased the biomass of Asian cabbage (Zhou et al. 2021). However, in a different scenario, the effect of IAA on root elongation could be self-defeating, and lead to increased metal absorption (Montreemuk et al. 2024; Wu et al. 2020). In both cases, the study of IAA production by bacteria with MICP activity could help explain the results found in field or nursery applications to mitigate the presence of the metal in plants and could be used to establish an appropriate selection strategy according to the soil-plant system of interest.

The application of bacteria for bioremediation purposes could be limited by the effect of Cd in enzymatic activities. Both effects (reduction and increase) of Cd on ureolytic activity have been reported in the literature. For example, the ureolytic activity of *Enterobacter hormaechei* IITISM-SA3 decreased (54%) when the metal concentration increased from 0 to 50 mg L^− 1^; however, no significant differences were observed between 0 mg L^− 1^ (control) and 7 mg L^− 1^ (Anand et al. 2024). This is like the report for *Enterobacter* sp. CJW-1, where a high increase in Cd concentration from 20 to 100 mg L^− 1^ decreased the ureolytic activity of the bacteria (Peng et al. 2020). On the other hand, like our results for *Serratia* sp. 89a, the presence of cadmium (0.05 mM) stimulated the ureolytic activity of *Serratia* sp. 4.1a and *Serratia* sp. 5b (Diez-Marulanda and Brandão 2023). Conversely, a decrease in ureolytic activity was reported for *Serratia marcescens* NCIM2919 when the Cd concentration increased from 5 to 15 mg L^− 1^ (Bhattacharya et al. 2018). The above indicates that the effect of Cd on ureolytic activity could be dosage-dependent, and future experiments changing Cd concentration (based on application necessities) should be implemented. The negative effect of Cd on ureolytic activity has been attributed to decreased bacterial growth (Peng et al. 2020), and the inhibition in the expression of *ure*C, *ure*E, *ure*F, and *ure*G genes (Zeng et al. 2022). It is important to mention that Ca addition in the MICP medium alleviated Cd-induced toxicity and reduced the negative impact on ureolytic activity (Fang et al. 2021). Some bacteria showed increased ureolytic activity in the presence of Cd. This could be a bacterial response to the stress caused by the metal, which induces Cd mineralization to reduce its toxicity (Huang et al. 2022). In this study, Cd does not have an adversed effect on some bacteria (e.g., *Bacillus* sp. 85d and *Serratia* sp. 89a), representing an advantage for future applications.

Related to the impact of Cd in IAA production, it has been reported for *B. subtilis* NA2 that the presence of Cd (800 mg L^− 1^) decreased IAA levels from 23.46 mg L^− 1^ to 14.49 mg L^− 1^ (Bashir et al. 2022). Likewise, in our study, IAA concentration decreased from 2.3 mg L^− 1^ CFU^− 1^ to 0.8 mg L^− 1^ CFU^− 1^ for *Bacillus* sp. 85d in the presence of Cd. Furthermore, Zhou et al. (2021) analyzed the IAA production for *Comamonas testoteroni* ZG2 at Cd concentrations of 0, 10, 20 and 40 mg L^− 1^, and reported that only at 40 mg L^− 1^ a reduction was observed; for the lowest concentrations, the authors indicated a slight increase in IAA production. It is also noteworthy that 60 mg L^− 1^ Cd does not significatively affect IAA production of *Comamonas* sp. 76 f. This indicates that different Cd concentrations need to be considered to assess the effect of Cd on IAA production, considering future application particularities. It has been reported that 50 mg L^− 1^ of Cd decreased IAA production for two bacteria of the *Serratia* genus (Carlos et al. 2016), which differ from our results where no effect was observed for *Serratia* sp. 89a. Contrary to our results, the presence of Cd (0.4 mM) stimulated IAA production in strains *P. fluorescens* ACC9 and *P. tolaasii* ACC23 (Dell’Amico et al. 2008). Also, *Klebsiella* sp. Mc173 strain showed an increase in IAA production in the presence of 50 mg L^− 1^ of Cd (Carlos et al. 2016). Like the negative effect of Cd on ureolytic activity, the decrease in IAA production could be associated with decreased bacterial growth in the presence of Cd; however, correction with CFU was used to compensate this aspect, so further studies (e.g., at the molecular level) should be performed to understand the negative effect of Cd in IAA production. On the other hand, some authors have reported the involvement of siderophores to mitigate IAA production inhibition by metals such as Cd (Chen et al. 2016; Dimkpa et al. 2008).

Various isolates showed higher Cd removal compared to other bacteria reported in the literature (Supplementary Table S8). Under the experimental conditions tested, *Serratia* sp. 89a and *Pseudomonas* sp. 85f showed higher Cd removal than other bacteria of the same genus, such as *Serratia* sp 4.1a and *Serratia* sp. 5b (> 99% removal at 17 mg L^− 1^ Cd after 69–120 h) (Diez-Marulanda and Brandão 2024), *Serratia marcescens* NCIM2919 (65% at 15 mg L^− 1^ after 96 h) (Bhattacharya et al. 2018), and *Pseudomonas aeruginosa* QD5 and QZ9 (≈ 85% at ≈ 11 mg L^− 1^ after 5 days) (Al Disi et al. 2022), all of which involved longer incubation periods and lower initial Cd concentrations than those used in this study. Other isolates showed lower performance compared to the literature, though that could be associated with incubation times and amounts of urea and Ca used (Liu et al. 2023), which were not optimized in this work. To the best of our knowledge, cadmium removal by MICP for *Delftia*, *Flavobacterium*, and *Klebsiella* genera is reported here for the first time. In a previous study, where Cd was precipitated using anaerobic granular sludge, CdCO_3_ formation was associated with *Klebsiella pneumoniae* (Martínez et al. 2020); however, the authors did not report its direct involvement during Cd precipitation by MICP.

Interestingly, the bacteria with greater Cd removal did not correspond to the bacteria with greater ureolytic activity. The relationship between ureolytic activity and Cd removal, showed a moderate positive correlation (R^2^ = 0.3908) (Supplementary Fig. S4), indicating that other aspects must be considered to explain the percentage of Cd removed. Several forms of interaction between bacteria and Cd have been reported, such as biosorption and bioaccumulation (Bravo and Braissant 2022). In a recent study on different dynamic micro-processes during a MICP assay, it was reported that 3.97%, 36.35%, and 49.27% of the removed Cd was accumulated inside the cells, adsorbed by the cells, and associated with precipitates, respectively (Sheng et al. 2022). This indicates that, although Cd is mainly removed by precipitation, there is a relevant percentage associated with other metal-bacteria interactions. Thus, further studies are crucial to determine the contribution of MICP and other mechanisms (e.g., bioaccumulation, biosorption) in metal removal. In addition, it is essential to apply the selected isolates directly to soil to evaluate their performance during toxic element remediation. Considering that pseudo-total Cd concentrations of up to 27 mg kg^− 1^ have been reported in cacao-growing soils (Bravo et al. 2021), bacteria with at least 50% Cd removal could be considered for further soil bioremediation studies. We report 17 isolates with this feature; nonetheless, optimizing experimental conditions may improve the removal capacity of more bacteria.

## Conclusions

In this study, Cd-tolerant ureolytic bacteria were isolated from cacao rhizosphere soils from producing farms in Santander, Colombia. All isolates showed MICP activity and IAA production, indicating that these types of soils harbor bacteria with multiple functions to cope with Cd. Since 60 mg kg^− 1^ of Cd increased ureolytic activity and did not affect the IAA production of some isolates, the different known Cd concentrations of cacao-growing soils should not be a limiting factor in future soil treatments. Furthermore, strains of the genera *Serratia*,* Klebsiella*, and *Stenotrophomonas* were evidenced to have the highest removal percentages (> 80%). To the best of our knowledge, MICP activity for the *Delftia* genus and Cd removal by MICP for the genera *Delftia*, *Flavobacterium*, and *Klebsiella* are reported for the first time.

This research presents ureolytic bacteria with potential to be used in future soil bioremediation studies. Considering the IAA production found in the isolated bacteria with MICP activity, this work contributes to the development of comprehensive bioremediation strategies to tackle Cd absorption (via MICP) and its adverse effects on plant growth (via IAA production).

## Electronic supplementary material

Below is the link to the electronic supplementary material.

Supplementary Material 1

## Data Availability

No datasets were generated or analysed during the current study.

## References

[CR1] Achal V, Pan X, Zhang D (2011) Remediation of copper-contaminated soil by *Kocuria flava* CR1, based on microbially induced calcite precipitation. Ecol Eng 37(10):1601–1605. 10.1016/j.ecoleng.2011.06.008

[CR2] Adarme-Duran CA, Ágreda J, Brandão PFB, Castillo E (2024) Cadmium availability in rhizosphere and non-rhizosphere soils in Cacao farms in santander, Colombia. Environ Monit Assess 196(12):1254. 10.1007/s10661-024-13301-x 39589552 10.1007/s10661-024-13301-x PMC11599408

[CR3] Al Disi Z, Attia E, Ahmad MI, Zouari N (2022) Immobilization of heavy metals by microbially induced carbonate precipitation using hydrocarbon-degrading ureolytic bacteria. Biotechnol Rep 35:e00747. 10.1016/j.btre.2022.e00747 10.1016/j.btre.2022.e00747 PMC9218142 35755319

[CR4] Anand S, Kumar V, Singh A, Phukan D, Pandey N (2024) Statistical modelling, optimization, and mechanistic exploration of novel ureolytic *Enterobacter hormaechei* IITISM-SA3 in cadmium immobilization under microbial inclusive and cell-free conditions through microbially induced calcite precipitation. Environ Pollut 348:123880. 10.1016/j.envpol.2024.123880 38554835 10.1016/j.envpol.2024.123880

[CR5] Bashir S, Javed S, Al-Anazi KM, Farah MA, Ali S (2022) Bioremediation of cadmium toxicity in wheat (Triticum aestivum L.) plants primed with L-Proline, Bacillus subtilis and Aspergillus Niger. Int J Environ Res Public Health 19(19):12683. 10.3390/ijerph191912683 36231984 10.3390/ijerph191912683 PMC9564855

[CR6] Bassam BJ, Caetano-Anollés G, Gresshoff PM (1991) Fast and sensitive silver staining of DNA in polyacrylamide gels. Anal Biochem 196(1):80–83. 10.1016/0003-2697(91)90120-I 1716076 10.1016/0003-2697(91)90120-i

[CR7] Bhattacharya A, Naik SN, Khare SK (2018) Harnessing the bio-mineralization ability of urease producing Serratia marcescens and Enterobacter cloacae EMB19 for remediation of heavy metal cadmium (II). J Environ Manage 215:143–152. 10.1016/j.jenvman.2018.03.055 29567554 10.1016/j.jenvman.2018.03.055

[CR8] Bibi S, Oualha M, Ashfaq MY, Suleiman MT, Zouari N (2018) Isolation, differentiation and biodiversity of ureolytic bacteria of Qatari soil and their potential in microbially induced calcite precipitation (MICP) for soil stabilization. RSC Adv 8(11):5854–5863. 10.1039/C7RA12758H 35539599 10.1039/c7ra12758h PMC9078176

[CR9] Brandão PFB, Torimura M, Kurane R, Bull AT (2002) Dereplication for biotechnology screening: PyMS analysis and PCR-RFLP-SSCP (PRS) profiling of 16S rRNA genes of marine and terrestrial actinomycetes. Appl Microbiol Biotechnol 58(1):77–83. 10.1007/s00253-001-0855-x 11833532 10.1007/s00253-001-0855-x

[CR10] Bravo D, Braissant O (2022) Cadmium-tolerant bacteria: current trends and applications in agriculture. Lett Appl Microbiol 74(3):311–333. 10.1111/lam.13594 34714944 10.1111/lam.13594 PMC9299123

[CR11] Bravo D, Leon-Moreno C, Martinez CA, Varon-Ramirez VM, Araujo-Carrillo GA, Vargas R, Quiroga-Mateus R, Zamora A, Rodriguez EAG (2021) The first National survey of cadmium in Cacao farm soil in Colombia. Agronomy 11(4):761. 10.3390/agronomy11040761

[CR12] Breakwell D, MacDonald B, Woolverton C, Smith K, Robison R (2007) Colony morphology protocol. American society for microbiology, 1–7. Available online: https://asm.org/ASM/media/Protocol-Images/Colony-Morphology-Protocol.pdf?ext=.pdf

[CR13] Bunsangiam S, Thongpae N, Limtong S, Srisuk N (2021) Large scale production of indole-3-acetic acid and evaluation of the inhibitory effect of indole-3-acetic acid on weed growth. Sci Rep 11(1):13094. 10.1038/s41598-021-92305-w 34158557 10.1038/s41598-021-92305-w PMC8219710

[CR14] Cai Q, Xu M, Ma J, Zhang X, Yang G, Long L, Chen C, Wu J, Song C, Xiao Y (2023) Improvement of cadmium immobilization in contaminated paddy soil by using ureolytic bacteria and rice straw. Sci Total Environ 874:162594. 10.1016/j.scitotenv.2023.162594 36870501 10.1016/j.scitotenv.2023.162594

[CR15] Carlos MHJ, Stefani PVY, Janette AM, Melani MSS, Gabriela PO (2016) Assessing the effects of heavy metals in ACC deaminase and IAA production on plant growth-promoting bacteria. Microbiol Res 188:53–61. 10.1016/j.micres.2016.05.001 27296962 10.1016/j.micres.2016.05.001

[CR16] Chen Y, Chao Y, Li Y, Lin Q, Bai J, Tang L, Wang S, Ying R, Qiu R (2016) Survival strategies of the plant-associated bacterium *Enterobacter* sp. strain EG16 under cadmium stress. Appl Environ Microbiol 82(6):1734–1744. 10.1128/AEM.03689-15 26729719 10.1128/AEM.03689-15 PMC4784038

[CR17] Cheng C, Han H, Wang Y, He L, Sheng X (2020) Metal-immobilizing and urease-producing bacteria increase the biomass and reduce metal accumulation in potato tubers under field conditions. Ecotoxicol Environ Saf 203:111017. 10.1016/j.ecoenv.2020.111017 32678748 10.1016/j.ecoenv.2020.111017

[CR18] Christensen WB (1946) Urea decomposition as a means of differentiating Proteus and paracolon cultures from each other and from Salmonella and Shigella types. J Bacteriol 52(4):461–466 16561200 10.1128/jb.52.4.461-466.1946 PMC518212

[CR19] Cumming G, Fidler F, Vaux DL (2007) Error bars in experimental biology. J Cell Biol 177(1):7–11. 10.1128/jb.52.4.461-466.1946 17420288 10.1083/jcb.200611141 PMC2064100

[CR20] Darriba D, Taboada GL, Doallo R, Posada D (2012) jModelTest 2: more models, new heuristics and parallel computing. Nat Methods 9:772. 10.1038/nmeth.2109 22847109 10.1038/nmeth.2109 PMC4594756

[CR21] Dell’Amico E, Cavalca L, Andreoni V (2008) Improvement of *Brassica napus* growth under cadmium stress by cadmium-resistant rhizobacteria. Soil Biol Biochem 40(1):74–84. 10.1016/j.soilbio.2007.06.024

[CR22] Diez-Marulanda JC, Brandão PFB (2023) Isolation of urease-producing bacteria from cocoa farms soils in santander, colombia, for cadmium remediation. 3 Biotech 13(3):98. 10.1007/s13205-023-03495-1 36860360 10.1007/s13205-023-03495-1 PMC9968674

[CR23] Diez-Marulanda JC, Brandão PFB (2024) Potential use of two *Serratia* strains for cadmium remediation based on microbiologically induced carbonate precipitation and their cadmium resistance. Environ Sci Pollut Res 31(4):5319–5330. 10.1007/s11356-023-31062-x 10.1007/s11356-023-31062-x 38114705

[CR24] Dimkpa CO, Svatoš A, Dabrowska P, Schmidt A, Boland W, Kothe E (2008) Involvement of siderophores in the reduction of metal-induced Inhibition of auxin synthesis in Streptomyces spp. Chemosphere 74(1):19–25. 10.1016/j.chemosphere.2008.09.079 18986679 10.1016/j.chemosphere.2008.09.079

[CR25] Duan C, Yu XY, Yao XW, Zhu JH, Li GY (2023) Coupling reinforcement of uranium tailings via *Klebsiella*-induced calcium carbonate precipitation and waterborne polyurethane. Constr Build Mater 400:132641. 10.1016/j.conbuildmat.2023.132641

[CR26] Edgar RC (2004) MUSCLE: multiple sequence alignment with high accuracy and high throughput. Nucleic Acids Res 32(5):1792–1797. 10.1093/nar/gkh340 15034147 10.1093/nar/gkh340 PMC390337

[CR27] Etesami H (2018) Bacterial mediated alleviation of heavy metal stress and decreased accumulation of metals in plant tissues: mechanisms and future prospects. Ecotoxicol Environ Saf 147:175–191. 10.1016/j.ecoenv.2017.08.032 28843189 10.1016/j.ecoenv.2017.08.032

[CR28] Fang L, Niu Q, Cheng L, Jiang J, Yu YY, Chu J, Achal V, You T (2021) Ca-mediated alleviation of Cd^2+^ induced toxicity and improved Cd^2+^ biomineralization by *Sporosarcina pasteurii*. Sci Total Environ 787:147627. 10.1016/j.scitotenv.2021.147627

[CR29] Ferrer MR, Quevedo-Sarmiento J, Rivadeneyra MA, Bejar V, Delgado R, Ramos-Cormenzana A (1988) Calcium carbonate precipitation by two groups of moderately halophilic microorganisms at different temperatures and salt concentrations. Curr Microbiol 17:221–227. 10.1007/BF01589456

[CR30] Gang S, Sharma S, Saraf M, Buck M, Schumacher J (2019) Analysis of indole-3-acetic acid (IAA) production in *Klebsiella* by LC-MS/MS and the Salkowski method. Bio-protocol 9(9):e3230–e3230. 10.21769/BioProtoc.3230 33655016 10.21769/BioProtoc.3230 PMC7854044

[CR31] Genchi G, Sinicropi MS, Lauria G, Carocci A, Catalano A (2020) The effects of cadmium toxicity. Int J Environ Res Public Health 17(11):3782. 10.3390/ijerph17113782 32466586 10.3390/ijerph17113782 PMC7312803

[CR32] Guindon S, Gascuel O (2003) A simple, fast, and accurate algorithm to estimate large phylogenies by maximum likelihood. Syst Biol 52(5):696–704. 10.1080/10635150390235520 14530136 10.1080/10635150390235520

[CR33] Halim MA, Rahman MM, Megharaj M, Naidu R (2020) Cadmium immobilization in the rhizosphere and plant cellular detoxification: role of plant-growth-promoting rhizobacteria as a sustainable solution. J Agric Food Chem 68(47):13497–13529. 10.1021/acs.jafc.0c04579 33170689 10.1021/acs.jafc.0c04579

[CR34] Hall TA (1999) Bioedit: a user-friendly biological sequence alignment editor and analysis program for windows 95/98/nt. In Nucleic acids symposium series 41(41), 95–98

[CR36] Han H, Wang Q, He LY, Sheng XF (2018) Increased biomass and reduced rapeseed cd accumulation of oilseed rape in the presence of cd-immobilizing and polyamine-producing bacteria. J Hazard Mater 353:280–289. 10.1016/j.jhazmat.2018.04.024 29677530 10.1016/j.jhazmat.2018.04.024

[CR35] Han H, Cai H, Wang X, Hu X, Chen Z, Yao L (2020) Heavy metal-immobilizing bacteria increase the biomass and reduce the cd and Pb uptake by Pakchoi (*Brassica chinensis* L.) in heavy metal-contaminated soil. Ecotoxicol Environ Saf 195:110375. 10.1016/j.ecoenv.2020.110375 32200142 10.1016/j.ecoenv.2020.110375

[CR37] Huang S, Liu R, Sun M, Li X, Guan Y, Lian B (2022) Transcriptome expression analysis of the gene regulation mechanism of bacterial mineralization tolerance to high concentrations of Cd^2+^. Sci Total Environ 806:150911. 10.1016/j.scitotenv.2021.150911 34653453 10.1016/j.scitotenv.2021.150911

[CR38] Jalilvand N, Akhgar A, Alikhani HA, Rahmani HA, Rejali F (2019) Removal of heavy metals zinc, lead, and cadmium by biomineralization of urease-producing bacteria isolated from Iranian mine calcareous soils. J Soil Sci Plant Nutr 20:206–219. 10.1007/s42729-019-00121-z

[CR39] Kato C, Li L, Tamaoka J, Horikoshi K (1997) Molecular analyses of the sediment of the 11000-m deep Mariana trench. Extremophiles 1(3):117–123. 10.1007/s007920050024 9680317 10.1007/s007920050024

[CR41] Kubier A, Wilkin RT, Pichler T (2019) Cadmium in soils and groundwater: A review. Appl Geochem 108:104388. 10.1016/j.apgeochem.2019.104388 10.1016/j.apgeochem.2019.104388 PMC7147761 32280158

[CR43] Kumar S, Stecher G, Li M, Knyaz C, Tamura K (2018) Mol Biol Evol 35(6):1547–1549. 10.1093/molbev/msy096. MEGA X: molecular evolutionary genetics analysis across computing platforms 10.1093/molbev/msy096 PMC5967553 29722887

[CR42] Kumar A, Song HW, Mishra S, Zhang W, Zhang YL, Zhang QR, Yu ZG (2023) Application of microbial-induced carbonate precipitation (MICP) techniques to remove heavy metal in the natural environment: A critical review. Chemosphere 318:137894. 10.1016/j.chemosphere.2023.137894 36657570 10.1016/j.chemosphere.2023.137894

[CR44] Kuzyakov Y, Razavi BS (2019) Rhizosphere size and shape: Temporal dynamics and Spatial stationarity. Soil Biol Biochem 135:343–360. 10.1016/j.soilbio.2019.05.011

[CR46] Li W, Yang Y, Achal V (2022) Biochemical composite material using corncob powder as a carrier material for ureolytic bacteria in soil cadmium immobilization. Sci Total Environ 802:149802. 10.1016/j.scitotenv.2021.149802 34464799 10.1016/j.scitotenv.2021.149802

[CR47] Liu P, Zhang Y, Tang Q, Shi S (2021) Bioremediation of metal-contaminated soils by microbially-induced carbonate precipitation and its effects on ecotoxicity and long-term stability. Biochem Eng J 166:107856. 10.1016/j.bej.2020.107856

[CR48] Liu Y, Ali A, Su JF, Li K, Hu RZ, Wang Z (2023) Microbial-induced calcium carbonate precipitation: influencing factors, nucleation pathways, and application in waste water remediation. Sci Total Environ 860:160439. 10.1016/j.scitotenv.2022.160439 36574549 10.1016/j.scitotenv.2022.160439

[CR49] Martínez CM, Rivera-Hernández M, Álvarez LH, Acosta-Rodríguez I, Ruíz F, Compeán-García VD (2020) Biosynthesis and characterization of cadmium carbonate crystals by anaerobic granular sludge capable of precipitate cadmium. Mater Chem Phys 246:122797. 10.1016/j.matchemphys.2020.122797

[CR50] Montaño-Salazar SM, Lizarazo-Marriaga J, Brandão PFB (2018) Isolation and potential biocementation of calcite precipitation inducing Bacteria from Colombian buildings. Curr Microbiol 75:256–265. 10.1007/s00284-017-1373-0 29043388 10.1007/s00284-017-1373-0

[CR51] Montoya C, Márquez MA, López JM, Cuervo C (2005) Caracterización de cristales de calcita bioprecipitada por un aislamiento nativo de *Bacillus subtilis*. Revista Colombiana de Biotecnología, 7(2), 19–25. Available online: https://repositorio.unal.edu.co/handle/unal/22100

[CR52] Montreemuk J, Stewart TN, Prapagdee B (2024) Bacterial-assisted phytoremediation of heavy metals: concepts, current knowledge, and future directions. Environ Technol Innov 33:103488. 10.1016/j.eti.2023.103488

[CR53] Okyay TO, Nguyen HN, Castro SL, Rodrigues DF (2016) CO_2_ sequestration by ureolytic microbial consortia through microbially-induced calcite precipitation. Sci Total Environ 572:671–680. 10.1016/j.scitotenv.2016.06.199 27524723 10.1016/j.scitotenv.2016.06.199

[CR54] Peng D, Qiao S, Luo Y, Ma H, Zhang L, Hou S, Wu B, Xu H (2020) Performance of microbial induced carbonate precipitation for immobilizing cd in water and soil. J Hazard Mater 400:123116. 10.1016/j.jhazmat.2020.123116 32569980 10.1016/j.jhazmat.2020.123116

[CR55] Qiao S, Zeng G, Wang X, Dai C, Sheng M, Chen Q, Xu F, Xu H (2021) Multiple heavy metals immobilization based on microbially induced carbonate precipitation by ureolytic bacteria and the precipitation patterns exploration. Chemosphere 274:129661. 10.1016/j.chemosphere.2021.129661 33979921 10.1016/j.chemosphere.2021.129661

[CR56] R Core Team (2022) R: A language and environment for statistical computing. Version 4.2.2. R Foundation for Statistical Computing, Vienna, Austria. https://www.R-project.org/

[CR58] Ronquist F, Teslenko M, van der Mark P, Ayres DL, Darling A, Höhna S, Larget B, Liu L, Suchard MA, Huelsenbeck JP (2012) MrBAYES 3.2: efficient bayesian phylogenetic inference and model selection across a large model space. Syst Biol 61(3):539–542. 10.1093/sysbio/sys029 22357727 10.1093/sysbio/sys029 PMC3329765

[CR57] RStudio Team (2022) RStudio: integrated development environment for R. Version 2022.12.0353. RStudio. PBC, Boston, MA. https://www.rstudio.com/

[CR59] Sanders E, Miller K (2010) I, microbiologist: A discovery-based course in microbial ecology and molecular evolution. Washington D. C. ASM

[CR60] Schwieger F, Tebbe CC (1998) A new approach to utilize PCR-single-strand-conformation polymorphism for 16S rRNA gene-based microbial community analysis. Appl Environ Microbiol 64(12). 10.1128/aem.64.12.4870-4876.1998 10.1128/aem.64.12.4870-4876.1998 PMC90936 9835576

[CR61] Sheng M, Peng D, Luo S, Ni T, Luo H, Zhang R, Wen Y, Xu H (2022) Micro-dynamic process of cadmium removal by microbial induced carbonate precipitation. Environ Pollut 308:119585. 10.1016/j.envpol.2022.119585 35728693 10.1016/j.envpol.2022.119585

[CR62] Taharia M, Dey D, Das K, Sukul U, Chen JS, Banerjee P, Dey G, Sharma RK, Lin PY, Chen CY (2024) Microbial induced carbonate precipitation for remediation of heavy metals, ions and radioactive elements: A comprehensive exploration of prospective applications in water and soil treatment. Ecotoxicol Environ Saf 271:115990. 10.1016/j.ecoenv.2024.115990 38262090 10.1016/j.ecoenv.2024.115990

[CR63] Tamayo-Figueroa DP, Castillo E, Brandão PFB (2019) Metal and metalloid immobilization by microbiologically induced carbonates precipitation. World J Microbiol Biotechnol 35:1–10. 10.1007/s11274-019-2626-9 10.1007/s11274-019-2626-9 30900009

[CR64] Torres-Aravena ÁE, Duarte-Nass C, Azócar L, Mella-Herrera R, Rivas M, Jeison D (2018) Can microbially induced calcite precipitation (MICP) through a ureolytic pathway be successfully applied for removing heavy metals from wastewaters? Crystals 8(11):438. 10.3390/cryst8110438

[CR65] Vanderschueren R, Argüello D, Blommaert H, Montalvo D, Barraza F, Maurice L, Schreck E, Lewis C, Vazquez JL, Umaharam P, Chavez E, Sarret G, Smolders E (2021) Mitigating the level of cadmium in Cacao products: reviewing the transfer of cadmium from soil to chocolate bar. Sci Total Environ 781:146779. 10.1016/j.scitotenv.2021.146779

[CR66] Verdouw H, Van Echteld CJA, Dekkers EMJ (1978) Ammonia determination based on indophenol formation with sodium salicylate. Water Res 12(6):399–402. 10.1016/0043-1354(78)90107-0

[CR67] Wang T, Wang S, Tang X, Fan X, Yang S, Yao L, Li Y, Han H (2020) Isolation of urease-producing bacteria and their effects on reducing cd and Pb accumulation in lettuce (*Lactuca sativa* L). Environ Sci Pollut Res 27:8707–8718. 10.1007/s11356-019-06957-3 10.1007/s11356-019-06957-3 31912394

[CR68] Wei T, Li H, Yashir N, Li X, Jia H, Ren X, Yang J, Hua L (2022a) Effects of urease-producing bacteria and eggshell on physiological characteristics and cd accumulation of Pakchoi (*Brassica chinensis* L.) plants. Environ Sci Pollut Res 29(42):63886–63897. 10.1007/s11356-022-20344-5 10.1007/s11356-022-20344-5 35469379

[CR69] Wei T, Yashir N, An F, Imtiaz SA, Li X, Li H (2022b) Study on the performance of carbonate-mineralized bacteria combined with eggshell for immobilizing Pb and cd in water and soil. Environ Sci Pollut Res 29:2924–2935. 10.1007/s11356-021-15138-0 10.1007/s11356-021-15138-0 34382171

[CR70] Wu Y, Ma L, Liu Q, Vestergård M, Topalovic O, Wang Q, Zhou Q, Huang L, Yang X, Feng Y (2020) The plant-growth promoting bacteria promote cadmium uptake by inducing a hormonal crosstalk and lateral root formation in a hyperaccumulator plant *Sedum Alfredii*. J Hazard Mater 395:122661. 10.1016/j.jhazmat.2020.122661 32305720 10.1016/j.jhazmat.2020.122661

[CR71] Yan ZX, Li Y, Peng SY, Wei L, Zhang B, Deng XY, Zhong M, Cheng X (2024) Cadmium biosorption and mechanism investigation using two cadmium-tolerant microorganisms isolated from rhizosphere soil of rice. J Hazard Mater 470:134134. 10.1016/j.jhazmat.2024.134134 38554514 10.1016/j.jhazmat.2024.134134

[CR72] Yang J, Jiang L, Guo Z, Sarkodie EK, Li K, Shi J, Peng Y, Liu H, Liu X (2024) The cd immobilization mechanisms in paddy soil through ureolysis-based microbial induced carbonate precipitation: emphasis on the coexisting cations and metatranscriptome analysis. J Hazard Mater 465:133174. 10.1016/j.jhazmat.2023.133174 38086299 10.1016/j.jhazmat.2023.133174

[CR73] Zeng Y, Chen Z, Lyu Q, Wang X, Du Y, Huan C, Liu Y, Yan Z (2022) Mechanism of microbiologically induced calcite precipitation for cadmium mineralization. Sci Total Environ 852:158465. 10.1016/j.scitotenv.2022.158465 36063935 10.1016/j.scitotenv.2022.158465

[CR74] Zhang W, Zhang H, Xu R, Qin H, Liu H, Zhao K (2023) Heavy metal bioremediation using microbially induced carbonate precipitation: key factors and enhancement strategies. Front Microbiol 14:1116970. 10.3389/fmicb.2023.1116970 36819016 10.3389/fmicb.2023.1116970 PMC9932936

[CR75] Zheng Y, Xiao C, Chi R (2021) Remediation of soil cadmium pollution by biomineralization using microbial-induced precipitation: a review. World J Microbiol Biotechnol 37(12):208. 10.1007/s11274-021-03176-2 34719751 10.1007/s11274-021-03176-2

[CR76] Zhou X, Yang Y, Yin Q, Zhang X, Li M (2021) Application potential of *Comamonas testosteroni* ZG2 for vegetable cultivation in nickel and cadmium polluted soil. Environ Technol Innov 23:101626. 10.1016/j.eti.2021.101626

[CR77] Zhu X, Li W, Zhan L, Huang M, Zhang Q, Achal V (2016) The large-scale process of microbial carbonate precipitation for nickel remediation from an industrial soil. Environ Pollut 219:149–155. 10.1016/j.envpol.2016.10.047 27814530 10.1016/j.envpol.2016.10.047

