# Supplementary material for: Cadmium removal and indole acetic acid production by ureolytic bacteria isolated from rhizosphere soils
Source: World J Microbiol Biotechnol. 2025 Aug 8;41(8):302. doi: 10.1007/s11274-025-04482-9 (PMC12334442; doi:10.1007/s11274-025-04482-9)
Supplement: Supplementary file 1 — Supplementary Material 1 [file 11274_2025_4482_MOESM1_ESM.docx]

**CADMIUM REMOVAL AND INDOLE ACETIC ACID PRODUCTION BY UREOLYTIC BACTERIA ISOLATED FROM RHIZOSPHERE SOILS**

**
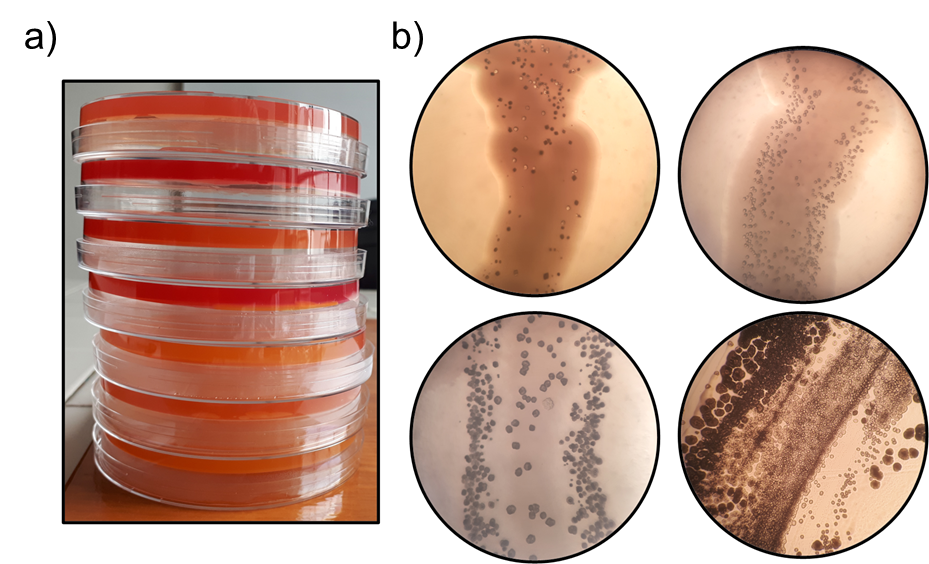
**

**Fig. S1.** Qualitative differences of **a)** ureolytic (with different color intensities) and **b)** MICP activities (precipitates with different size and shape) of selected bacteria isolated in the study. The precipitates were observed with an optical microscope (CX31 Upright Microscope, Olympus) and photographs were taken with a cell phone.


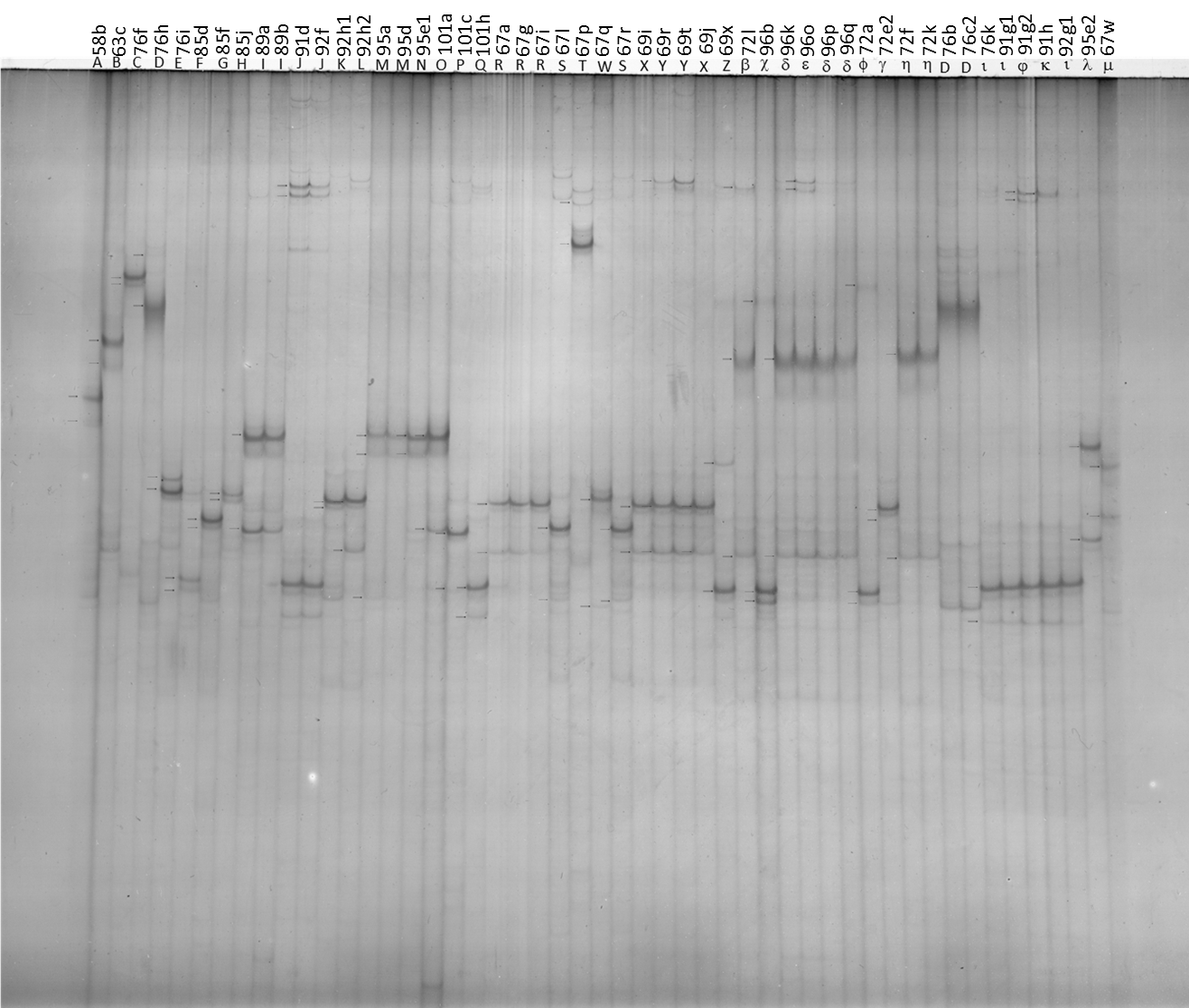


**Fig. S2**. SSCP profiles of the 16S rRNA gene V4-V5 regions of isolates from cocoa rhizospheric soils. Each lane indicates the code of the strain evaluated. Capital letters and symbols indicates a unique SSCP pattern.


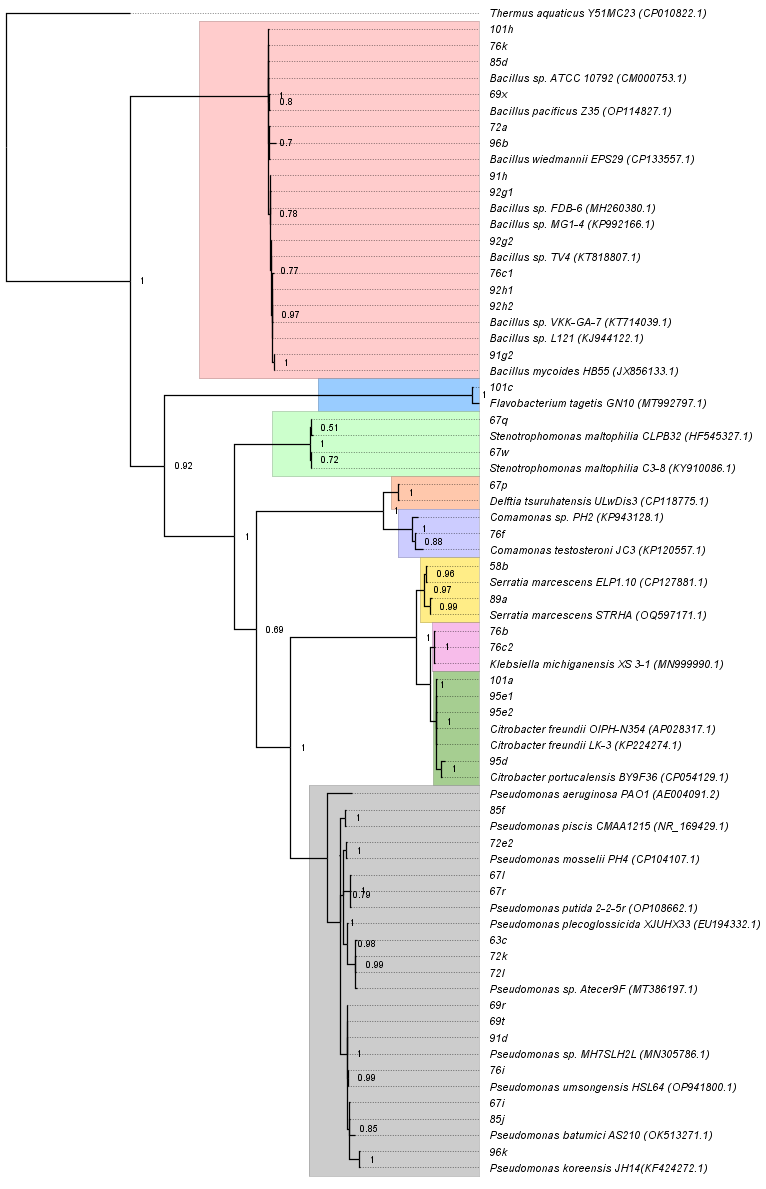


**Fig. S3.** Phylogenetic tree of the isolates obtained from rhizospheric cocoa soils, based on the 16S rRNA gene. The colors indicate different clades that correspond to the 9 genera previously identified by BLAST. The numbers on the branches are the posterior probability.


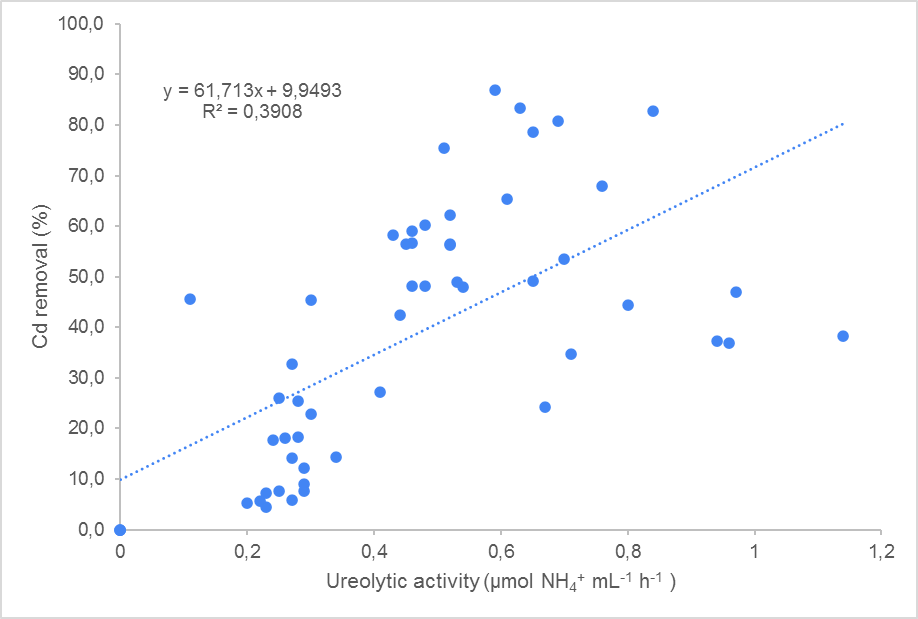


**Fig. S4.** Correlation between ureolytic activity and Cd removal of bacteria isolated from rhizosphere soils of cacao. The R^2^ and regression equation are stated.

**Table S1.** Primers used for SSCP analysis and molecular identification of ureolytic isolates*.*

| **Primer** | **Sequence (5’ - 3’)** | **Annealing temperature (°C)** | **Amplicon size**  **(bp)** | **Reference** |
| --- | --- | --- | --- | --- |
| 519F | CAGCMGCCGCGGTAATAC | 55 | ~400 | Schwieger & Tebbe, 1998 |
| 909R | CCGTCAATTCATTTGAGT |  |  | Kato et al., 1997 |
| 27F | AGAGTTTGATCMTGGCTCAG | 55 | ~1500 | Weisburg et al., 1991 |
| 1492R | GGTTACCTTGTTACGACTT |  |  |  |

**Table S2.** Master Mix for PCR reactions.

| **Master Mix**  **reagent** | **Stock solution**  **concentration** | **Final**  **concentration** |
| --- | --- | --- |
| Buffer | 10X | 1x |
| Mg^2+^ | 50 mM | 1.5 mM |
| dNTP | 10 mM | 0.2 mM |
| primer 1492R or 909R (V4-V5 regions) | 10 mM | 0.4 mM |
| primer 27F or 519F (16S rRNA) | 10 mM | 0.4 mM |
| Taq Polimerase | 5 U/µL | 1 U/µL |

**Table** **S3**. Macroscopic morphological characterization of ureolytic bacteria with MICP activity isolated from rhizospheric cacao soils in culture medium with Cd (5 mg kg^-1^).

| **#** | **Strain code** | **Size** | **Form** | **Margin** | **Opacity** | **Color** | **Elevation** | **Texture** |
| --- | --- | --- | --- | --- | --- | --- | --- | --- |
| 1 | 58b | Medium | Irregular | Undulate | Opaque | Creamy | Convex | Smooth |
| 2 | 63c | Small | Circular | Entire | Opaque | Creamy | Flat | Smooth |
| 3 | 76f | Small | Irregular | Undulate | Translucent | Creamy | Flat | Mucoid |
| 4 | 76h | Medium | Circular | Entire | Opaque | Creamy | Convex | Mucoid |
| 5 | 76i | Small | Irregular | Undulate | Opaque | Creamy | Convex | Mucoid |
| 6 | 85d | Small | Circular | Entire | Translucent | Creamy | Flat | Mucoid |
| 7 | 85f | Punctiform | Undetermined | Undetermined | Undetermined | Creamy | Undetermined | Mucoid |
| 8 | 85j | Punctiform | Undetermined | Undetermined | Undetermined | Creamy | Undetermined | Mucoid |
| 9 | 89a | Medium | Circular | Entire | Opaque | Red/Orange | Convex | Smooth |
| 10 | 89b | Medium | Circular | Entire | Opaque | Creamy | Convex | Smooth |
| 11 | 91d | Punctiform | Undetermined | Undetermined | Undetermined | Creamy | Undetermined | Mucoid |
| 12 | 92f | Small | Irregular | Undulate | Opaque | Creamy | Flat | Smooth |
| 13 | 92h1 | Medium | Circular | Entire | Opaque | Creamy | Convex | Smooth |
| 14 | 92h2 | Large | Irregular | Undulate | Opaque | Creamy | Convex | Mucoid |
| 15 | 95a | Large | Irregular | Undulate | Translucent | Creamy | Convex | Smooth |
| 16 | 95d | Large | Irregular | Lobate | Translucent | Creamy | Convex | Smooth |
| 17 | 95e1 | Small | Irregular | Undulate | Opaque | Creamy | Convex | Mucoid |
| 18 | 101a | Medium | Circular | Entire | Opaque | Creamy | Convex | Smooth |
| 19 | 101c | Punctiform | Undetermined | Undetermined | Undetermined | Undetermined | Undetermined | Smooth |
| 20 | 101h | Large | Irregular | Undulate | Opaque | Creamy | Convex | Mucoid |
| 21 | 67a | Small | Circular | Entire | Opaque | Creamy | Convex | Mucoid |
| 22 | 67g | Medium | Irregular | Entire | Opaque | Creamy | Convex | Mucoid |
| 23 | 67i | Large | Circular | Entire | Opaque | Creamy | Convex | Mucoid |
| 24 | 67l | Large | Circular | Undulate | Opaque | Creamy | Convex | Mucoid |
| 25 | 67p | Large | Irregular | Undulate | Translucent | Creamy | Flat | Mucoid |
| 26 | 67q | Small | Circular | Entire | Opaque | Yellow | Convex | Smooth |
| 27 | 67r | Large | Circular | Undulate | Opaque | Creamy | Convex | Mucoid |
| 28 | 69f | Medium | Circular | Entire | Opaque | Creamy | Convex | Smooth |
| 29 | 69i | Large | Irregular | Entire | Opaque | Creamy | Convex | Mucoid |
| 30 | 69j | Small | Circular | Entire | Opaque | Creamy | Convex | Mucoid |
| 31 | 69t | Medium | Circular | Entire | Opaque | Creamy | Convex | Mucoid |
| 32 | 69r | Medium | Circular | Entire | Opaque | Creamy | Convex | Mucoid |
| 33 | 69x | Large | Irregular | Undulate | Opaque | Creamy | Flat | Smooth |
| 34 | 72l | Large | Irregular | Undulate | Opaque | Creamy | Convex | Mucoid |
| 35 | 96b | Large | Irregular | Undulate | Opaque | Creamy | Pulvinate | Smooth |
| 36 | 96k | Large | Circular | Entire | Opaque | Creamy | Convex | Mucoid |
| 37 | 96o | Medium | Circular | Entire | Opaque | Creamy | Convex | Mucoid |
| 38 | 96p | Medium | Circular | Entire | Opaque | Creamy | Convex | Mucoid |
| 39 | 96q | Large | Irregular | Undulate | Opaque | Creamy | Convex | Mucoid |
| 40 | 72a | Small | Irregular | Undulate | Opaque | Creamy | Convex | Smooth |
| 41 | 72e2 | Small | Irregular | Undulate | Translucent | Yellow | Flat | Smooth |
| 42 | 72f | Large | Irregular | Entire | Opaque | Creamy | Convex | Mucoid |
| 43 | 72k | Small | Irregular | Undulate | Opaque | Creamy | Convex | Mucoid |
| 44 | 76b | Small | Circular | Entire | Opaque | Creamy | Convex | Mucoid |
| 45 | 76c1 | Medium | Irregular | Lobate | Opaque | Creamy | Convex | Smooth |
| 46 | 76c2 | Small | Circular | Entire | Opaque | Creamy | Convex | Mucoid |
| 47 | 76k | Large | Irregular | Undulate | Opaque | Yellow | Convex | Smooth |
| 48 | 91g1 | Medium | Irregular | Undulate | Opaque | Yellow | Convex | Mucoid |
| 49 | 91g2 | Large | Rhizoid | Rhizoid | Opaque | Creamy | Convex | Smooth |
| 50 | 91h | Medium | Circular | Entire | Opaque | Creamy | Convex | Smooth |
| 51 | 92g1 | Small | Circular | Entire | Opaque | Creamy | Convex | Smooth |
| 52 | 92g2 | Large | Irregular | Undulate | Opaque | Creamy | Convex | Smooth |
| 53 | 95e2 | Small | Irregular | Undulate | Opaque | Creamy | Convex | Mucoid |
| 54 | 67w | Medium | Circular | Entire | Opaque | Yellow | Convex | Mucoid |

**Table S4**. Gram stain and microscopic morphology of ureolytic bacteria with MICP activity isolated from rhizospheric cacao soils.

| **#** | **Strain code** | **Gram stain** | **Shape** | **#** | **Strain code** | **Gram stain** | **Shape** |
| --- | --- | --- | --- | --- | --- | --- | --- |
| 1 | 58b | - | Bacillus | 28 | 69f | + | Bacillus |
| 2 | 63c | - | Bacillus | 29 | 69i | - | Bacillus |
| 3 | 76f | - | Bacillus | 30 | 69j | + | Bacillus |
| 4 | 76h | - | Bacillus | 31 | 69t | - | Bacillus |
| 5 | 76i | - | Bacillus | 32 | 69x | + | Bacillus |
| 6 | 85d | + | Bacillus | 33 | 69r | - | Bacillus |
| 7 | 85f | - | Bacillus | 34 | 72l | - | Bacillus |
| 8 | 85j | - | Bacillus | 35 | 96b | + | Bacillus |
| 9 | 89a | - | Bacillus | 36 | 96o | - | Bacillus |
| 10 | 89b | - | Bacillus | 37 | 96p | - | Bacillus |
| 11 | 91d | - | Bacillus | 38 | 96q | - | Bacillus |
| 12 | 92f | - | Bacillus | 39 | 96k | - | Bacillus |
| 13 | 92h1 | + | Bacillus | 40 | 72a | + | Bacillus |
| 14 | 92h2 | + | Bacillus | 41 | 72e2 | - | Bacillus |
| 15 | 95a | - | Bacillus | 42 | 72f | - | Bacillus |
| 16 | 95d | - | Bacillus | 43 | 72k | - | Bacillus |
| 17 | 95e1 | - | Coccus | 44 | 76b | - | Bacillus |
| 18 | 101a | - | Bacillus | 45 | 76c1 | + | Bacillus |
| 19 | 101c | - | Bacillus | 46 | 76c2 | - | Bacillus |
| 20 | 101h | + | Bacillus | 47 | 76k | + | Bacillus |
| 21 | 67a | - | Bacillus | 48 | 91g1 | + | Bacillus |
| 22 | 67g | - | Bacillus | 49 | 91g2 | + | Bacillus |
| 23 | 67i | - | Bacillus | 50 | 91h | + | Bacillus |
| 24 | 67l | - | Bacillus | 51 | 92g1 | + | Bacillus |
| 25 | 67q | - | Bacillus | 52 | 92g2 | + | Bacillus |
| 26 | 67p | - | Bacillus | 53 | 95e2 | - | Bacillus |
| 27 | 67r | - | Bacillus | 54 | 67w | - | Bacillus |

**Table S5**. Identification of bacteria isolated from rhizospheric cacao soils in Santander - Colombia. Based on differentiated SSCP profiles and the characteristics analyzed, 40 isolates were selected to perform molecular identification by amplification of the 16S rRNA gene using primers 27F and 1492R.

| **Strain**  **Code** | **SSCP Profile Code** | **Identification*** | **Identity**  **(%)** | **Isolate GenBank Access number** | **GenBank**  **Access number of closest relative according to BLAST** |
| --- | --- | --- | --- | --- | --- |
| 58b | A | *Serratia marcescens* | 99 | PQ497376 | MN330072.1 |
| 63c | B | *Pseudomonas* sp. | 99 | PQ497377 | KY324893.1 |
| 76f | C | *Comamonas testosteroni* | 99 | PQ497394 | JQ361660.1 |
| 76b | D | *Klebsiella michiganensis* | 99 | PQ497391 | MN999990.1 |
| 76c2 | D | *Klebsiella michiganensis* | 99 | PQ497393 | MN999990.1 |
| 76i | E | *Pseudomonas* sp. | 99 | PQ497395 | OK274034.1 |
| 85d | F | *Bacillus toyonensis* | 100 | PQ497374 | MT605503.1 |
| 85f | G | *Pseudomonas* sp. *CMR5c* | 99 | PQ497373 | CP027705.1 |
| 85j | H | *Pseudomonas* sp. *BW7P1* | 99 | PQ497372 | CP103374.1 |
| 89a | I | *Serratia* sp. | 99 | PQ497371 | MK156456.1 |
| 101a | O | *Citrobacter* sp. | 100 | PQ497358 | CP047606.1 |
| 91d | J | *Pseudomonas* sp. *BW7P1* | 99 | PQ497370 | CP103374.1 |
| 92h1 | K | *Bacillus cereus* | 99 | PQ497365 | MT642947.1 |
| 92h2 | L | *Bacillus thuringiensis* | 99 | PQ497364 | CP044978.1 |
| 95d | M | *Citrobacter* sp. | 99 | PQ497363 | CP060662.1 |
| 95e1 | N | *Citrobacter* sp. | 99 | PQ497361 | CP042534.1 |
| 101c | P | *Flavobacterium* sp. | 99 | PQ497357 | JF772477.1 |
| 101h | Q | *Bacillus thuringiensis* | 100 | PQ497356 | MT534551.1 |
| 67i | R | *Pseudomonas* sp. *AM2(2011)* | 99 | PQ497378 | HQ600988.1 |
| 67l | S | *Pseudomonas fluorescens* | 99 | PQ497379 | MN585724.1 |
| 67r | S | *Pseudomonas fluorescens* | 100 | PQ497382 | MN585724.1 |
| 67p | T | *Delftia tsuruhatensis* | 100 | PQ497380 | MT374262.1 |
| 67q | W | *Stenotrophomonas* sp. *TD3* | 99 | PQ497381 | KP313737.1 |
| 69r | Y | *Pseudomonas* sp. *BW7P1* | 99 | PQ497384 | CP103374.1 |
| 69t | Y | *Pseudomonas* sp. *BW7P1* | 99 | PQ497385 | CP103374.1 |
| 69x | Z | *Bacillus pacificus* | 99 | PQ497386 | OP114834.1 |
| 72l | b | *Pseudomonas* sp. | 99 | PQ497390 | MT386197.1 |
| 96k | ẟ | *Pseudomonas* sp. | 100 | PQ497359 | MG491613.1 |
| 96b | 𝛘 | *Bacillus cereus* | 99 | PQ497360 | KC182060.1 |
| 72ª | ϕ | *Bacillus* sp. | 99 | PQ497387 | MT505107.1 |
| 72e2 | 𝜸 | *Pseudomonas mosselii* | 100 | PQ497388 | MK503435.1 |
| 72k | 𝛈 | *Pseudomonas* sp. | 99 | PQ497389 | MT386197.1 |
| 76k | 𝜄 | *Bacillus toyonensis* | 100 | PQ497375 | MT605501.1 |
| 92g1 | 𝜄 | *Bacillus* sp. *MG1-4* | 99 | PQ497367 | KP992166.1 |
| 91g2 | φ | *Bacillus mycoides* | 100 | PQ497369 | JX856133.1 |
| 91h | 𝜿 | *Bacillus proteolyticus* | 100 | PQ497368 | MT573794.1 |
| 95e2 | 𝛌 | *Citrobacter* sp. | 99 | PQ497362 | CP042534.1 |
| 67w | 𝛍 | *Stenotrophomonas maltophilia* | 100 | PQ497383 | KY910086.1 |
| 92g2 | - | *Bacillus cereus* | 100 | PQ497366 | KT818807.1 |
| 76c1 | - | *Bacillus thuringiensis* | 99 | PQ497392 | KT714039.1 |

* Name of the isolates with the highest percentage identity from the BLAST analysis.

**Table S6.** Genus assigned to some bacteria according to SSCP profile.

| **#** | **Strain Code** | **SSCP Profile Code** | **Putative identification** |
| --- | --- | --- | --- |
| 1 | 76h | D | *Klebsiella* sp. |
| 2 | 95a | M | *Citrobacter* sp. |
| 3 | 67a | R | *Pseudomonas* sp. |
| 4 | 67g | R | *Pseudomonas* sp. |
| 5 | 72f | 𝛈 | *Pseudomonas* sp. |
| 6 | 91g1 | 𝜄 | *Bacillus* sp. |
| 7 | 89b | I | *Serratia* sp. |

**Table S7.** Cd removal in aqueous solution by ureolytic bacteria isolated from rhizospheric soils.

| **#** | **Molecular identification** | **Strain code** | **Cd removal (%)** | **#** | **Molecular identification** | **Strain code** | **Cd removal (%)** |
| --- | --- | --- | --- | --- | --- | --- | --- |
| 1 | *Serratia* sp. | 89a | 87.0 | 27 | *Bacillus* sp. | 96b | 38.4 |
| 2 | *Klebsiella* sp.^b^ | 76h | 83.4 | 28 | *Bacillus* sp. | 92g1 | 37.4 |
| 3 | *Stenotrophomonas* sp. | 67w | 82.7 | 29 | *Bacillus* sp. | 91h | 36.9 |
| 4 | *Serratia* sp. | 58b | 80.8 | 30 | *Bacillus* sp. | 101h | 34.7 |
| 5 | *Serratia* sp. | 89b | 78.6 | 31 | *Delftia* sp. | 67p | 32.8 |
| 6 | *Comamonas* sp. | 76f | 75.5 | 32 | *Pseudomonas* sp.^b^ | 67a | 27.2 |
| 7 | *Bacillus* sp. | 76k | 68.0 | 33 | *Pseudomonas* sp. | 72l | 26.0 |
| 8 | *Bacillus* sp. | 85d | 65.4 | 34 | Unidentified^c^ | 96q | 25.4 |
| 9 | *Klebsiella* sp. | 76b | 62.2 | 35 | *Pseudomonas* sp. | 67l | 24.3 |
| 10 | *Citrobacter* sp. | 101a | 60.3 | 36 | *Pseudomonas* sp. | 96k | 22.8 |
| 11 | *Citrobacter* sp. | 95e1 | 59.1 | 37 | Unidentified^c^ | 96p | 18.3 |
| 12 | *Citrobacter* sp*.* | 95d | 58.2 | 38 | Unidentified^c^ | 96o | 18.2 |
| 13 | *Citrobacter* sp.^b^ | 95a | 56.6 | 39 | Unidentified^c^ | 69j | 17.8 |
| 14 | *Bacillus* sp. | 76c1 | 56.5 | 40 | *Bacillus* sp. | 92h2 | 14.3 |
| 15 | *Flavobacterium* sp. | 101c | 56.4 | 41 | *Pseudomonas* sp. | 67i | 14.1 |
| 16 | *Klebsiella* sp. | 76c2 | 56.2 | 42 | *Pseudomonas* sp.^b^ | 67g | 12.1 |
| 17 | *Bacillus* sp. | 92h1 | 53.5 | 43 | *Bacillus* sp. | 72a | 9.1 |
| 18 | *Stenotrophomonas* sp. | 67q | 49.2 | 44 | *Pseudomonas* sp. | 69t | 7.7 |
| 19 | *Pseudomonas* sp. | 85f | 48.9 | 45 | *Pseudomonas* sp. | 69r | 7.6 |
| 20 | *Pseudomonas* sp. | 92f | 48.2 | 46 | Unidentified^c^ | 69f | 7.3 |
| 21 | *Pseudomonas* sp. | 63c | 48.1 | 47 | *Pseudomonas* sp.^b^ | 72f | 5.9 |
| 22 | *Pseudomonas* sp. | 76i | 47.9 | 48 | *Pseudomonas* sp. | 72k | 5.7 |
| 23 | *Pseudomonas* sp. | 72e2 | 47.0 | 49 | Unidentified | 69i | 5.3 |
| 24 | *Pseudomonas* sp. | 91d | 45.5 | 50 | *Pseudomonas* sp. | 67r | 4.4 |
| 25 | *Pseudomonas* sp. | 85j | 45.3 | 51 | *Bacillus* sp. ^a^ | 91g1 | ND^a^ |
| 26 | *Bacillus* sp. | 69x | 44.4 | 52 | *Bacillus* sp.^a^ | 91g2 | ND^a^ |
| 27 | *Citrobacter* sp. | 95e2 | 42.4 | 54 | *Bacillus* sp.^a^ | 92g2 | ND^a^ |

^a^ ND: Not Determined. These strains did not grow in the medium used with 60 mg L^-1^ of Cd.

^b^ Putative identification according to SSCP profile.

^c^ Some isolates were not identified because they did not show traits with interest to this work.

**Table S8.** Comparison of Cd removal performance for a selection of isolates from this study with other bacteria reported in the literature.

|  | **This study^a^** | |  | **Literature** | | | |
| --- | --- | --- | --- | --- | --- | --- | --- |
| **Genus** | **Bacteria** | **Cd removal (%)^a^** |  | **Bacteria** | **Cd removal (%)** | **Experimental conditions** | **Reference** |
| *Serratia* | *Serratia* sp. 89a | 87.0 |  | *Serratia* sp. 4.1a y 5b | > 99 | 96 - 120h, Cd (17 mg L^-1^), urea (30 g L^-1^), Ca (6.80 mM). | Diez-Marulanda & Brandão, 2024 |
|  |  |  |  | *Serratia marcescens* | 65 | 96h, Cd (15 mg L^-1^), urea (2%), Ca (25 mM) | Bhattacharya et al., 2018 |
| *Stenotrophomonas* | *Stenotrophomonas* sp. 67w | 82.7 |  | *Stenotrophomonas rhizophila* | 71.3 | 96h, Cd (aprox. 224 mg L^-1^), urea (0.5 M), Ca (25 mM) | Jalilvand et al., 2019 |
| *Comamonas* | *Comamonas* sp. 76f | 75.5 |  | *Comamonas testosteroni ZG2* | 98.4 | 48h, aprox. 0.0119 M CdCl_2_ | Zhou et al., 2021 |
| *Bacillus* | *Bacillus* sp. 76k | 68.0 |  | *B. paramycoides* | 67.9 | 168h, Cd (aprox. 6.7 mg L^-1^), urea (2%), Ca (25 mM) | Kaur et al., 2024 |
|  |  |  |  | *Bacillus* sp. UR1, UR16 and UR21 | aprox. 25, 35 and 65 respectively | 72h, Cd (aprox. 224 mg L^-1^), urea (0.7 M) | Wei et al., 2022b |
| *Pseudomonas* | *Pseudomonas* 85f | 48.9 |  | *Pseudomonas aeruginosa* QD5 y QZ9 | aprox. 85 | 5 days, Cd (aprox. 11 mg L^-1^), urea (20 g L^-1^), Ca (0.1 M). | Al Disi et al., 2022 |
| *Citrobacter* | *Citrobacter* sp. 101a | 60.3 |  | *Citrobacter* sp. UR20 | 40 | 72h, Cd (aprox. 224 mg L^-1^), urea (0.7 M) | Wei et al., 2022b |

^a^ Experimental conditions for isolates of this study: LB medium supplemented with Cd(II) (60 mg L^-1^), urea (20 g L^-1^) and Ca(II) (1.2 g L^-1^, equivalent to ≈30 mM), with incubation for 48 h at 30 °C and 130 rpm.

**SUPPLEMENTARY MATERIAL REFERENCES**

Al Disi, Z., Attia, E., Ahmad, M. I., &amp; Zouari, N. (2022). Immobilization of heavy metals by microbially induced carbonate precipitation using hydrocarbon-degrading ureolytic bacteria. Biotechnology Reports, 35, e00747. https://doi.org/10.1016/j.btre.2022.e00747

Bhattacharya, A., Naik, S. N., &amp; Khare, S. K. (2018). Harnessing the bio-mineralization ability of urease producing Serratia marcescens and Enterobacter cloacae EMB19 for remediation of heavy metal cadmium (II). Journal of Environmental Management, 215, 143-152. https://doi.org/10.1016/j.jenvman.2018.03.055

Diez-Marulanda, J. C., &amp; Brandão, P. F. (2024). Potential use of two Serratia strains for cadmium remediation based on microbiologically induced carbonate precipitation and their cadmium resistance. Environmental Science and Pollution Research, 1-12. https://doi.org/10.1007/s11356-023-31062-x

Jalilvand, N., Akhgar, A., Alikhani, H. A., Rahmani, H. A., &amp; Rejali, F. (2019). Removal of heavy metals zinc, lead, and cadmium by biomineralization of urease-producing bacteria isolated from Iranian mine calcareous soils. Journal of Soil Science and Plant Nutrition, 20, 206-219. https://doi.org/10.1007/s42729-019-00121-z

Kato, C., Li, L., Tamaoka, J., & Horikoshi, K. (1997). Molecular analyses of the sediment of the 11000-m deep Mariana Trench. Extremophiles, 1(3), 117–123. https://doi.org/10.1007/s007920050024.

Kaur, M., Sidhu, N., &amp; Reddy, M. S. (2024). Removal of cadmium through biomineralization using halophilic and ureolytic bacteria under saline conditions. International Biodeterioration & Biodegradation, 191, 105805. https://doi.org/10.1016/j.ibiod.2024.105805

Schwieger, F., & Tebbe, C. C. (1998). A new approach to utilize PCR-single-strand-conformation polymorphism for 16S rRNA gene-based microbial community analysis. Applied and Environmental Microbiology, 64(12). https://doi.org/10.1128/aem.64.12.4870-4876.1998.

Wei, T., Yashir, N., An, F., Imtiaz, S. A., Li, X., &amp; Li, H. (2022b). Study on the performance of carbonate-mineralized bacteria combined with eggshell for immobilizing Pb and Cd in water and soil. Environmental Science and Pollution Research, 29, 2924-2935. https://doi.org/10.1007/s11356-021-15138-0

Weisburg, W. G., Barns, S. M., Pelletier, D. A., & Lane, D. J. (1991). 16S ribosomal DNA amplification for phylogenetic study. *Journal of Bacteriology*, *173*(2), 697-703. https://doi.org/10.1128/jb.173.2.697-703.1991

Zhou, X., Yang, Y., Yin, Q., Zhang, X., &amp; Li, M. (2021). Application potential of Comamonas testosteroni ZG2 for vegetable cultivation in nickel and cadmium polluted soil. Environmental Technology &amp; Innovation, 23, 101626. https://doi.org/10.1016/j.eti.2021.101626
